# Effects of varying levels of valine supplementation on meat metabolism and rumen microorganisms in Tibetan sheep

**DOI:** 10.3389/fmicb.2026.1806032

**Published:** 2026-03-27

**Authors:** Fuhong Wang, Lijuan Han, Shengzhen Hou, Linsheng Gui, Zhenzhen Yuan, Shengnan Sun, Chao Yang, Zhiyou Wang, Baochun Yang

**Affiliations:** College of Agriculture and Animal Husbandry, Qinghai University, Xining, China

**Keywords:** meat quality, metabolomics, rumen microbiota, Tibetan sheep, valine

## Abstract

**Introduction:**

This study seeks to examine the impact of supplementing different concentrations of valine (specifically, 0.1% and 0.15% treatment groups) on the rumen microbial community, muscle metabolites, and meat quality in Tibetan sheep.

**Methods:**

Both targeted and untargeted metabolomics approaches were employed to conduct a thorough analysis of meat quality and muscle metabolites. Simultaneously, 16S rDNA sequencing was utilized to explore the composition of the rumen microbial community in Tibetan sheep and its potential regulatory mechanisms on meat quality.

**Results:**

The findings reveal that the 0.15% valine treatment group exhibited superior edible quality and flavor characteristics, along with an increased protein content in the meat. In comparison to the control group (0% valine supplementation), the 0.1% and 0.15% treatment groups demonstrated an increase in meat yield by 2.24% and 3.61%, respectively, and a reduction in shear force by 18.33% and 26.93%. Correlation analysis between non-targeted metabolomics and meat quality indicated that valine supplementation may influence amino acid metabolism in muscle tissue, thereby affecting meat pH and tenderness. Furthermore, the supplementation of 0.15% valine led to an increased abundance of *Succiniclasticum*, *Christensenellaceae R-7 group*, and *NK4A214_group* in the rumen of Tibetan sheep, while concurrently reducing the relative abundance of certain uncultured bacterial taxa (e.g., *g__uncultured*).

**Discussion:**

Overall, in comparison to the 0% (K group) and 0.10% (V1 group) treatments, the 0.15% (V2 group) treatment resulted in enhanced meat quality and metabolite content. Additionally, valine supplementation exerted a significant impact on the composition of rumen microbial communities in Tibetan sheep, thereby may influence muscle tenderness and protein deposition.

## 1 Introduction

Qinghai Province, situated in western China on the northeastern periphery of the Qinghai-Tibet Plateau, is characterized by distinctive environmental conditions and serves as one of the primary habitats for Tibetan sheep in China. The Tibetan sheep, recognized as one of China’s three principal primitive sheep breeds, demonstrate remarkable adaptability to severe natural and ecological conditions, allowing them to flourish in high-altitude areas over extended duration (Sun et al., 2024). In comparison to other sheep breeds, Tibetan sheep meat is noted for its tenderness, high palatability, and mild in gamey taste, accompanied by a rich aroma and savory flavor. This makes it an exemplary animal-based product, distinguished by its high protein content, low fat, and substantial nutritional value (Liu et al., 2024). Tibetan sheep predominantly occupy high-altitude regions, where they are primarily reared through natural pasture grazing. The combination of relatively low external temperatures and prolonged periods of grass withering leads to an inadequate forage supply. Additionally, Qinghai’s delicate ecological environment is unable to support extensive grazing of Tibetan sheep, thereby limiting the development of the province’s Tibetan sheep industry. To effectively address this issue, the government is leading a transition from traditional grazing practices to standardized farming models for Qinghai’s Tibetan sheep.

Branched-chain amino acids (BCAAs), which include leucine (Leu), valine (Val), and isoleucine (Ile), have been shown to exert both direct and indirect metabolic effects, such as the regulation of body weight, muscle protein synthesis, energy homeostasis, feeding behavior, and glucose homeostasis (Tanase et al., 2024). BCAAs and their metabolites are involved in the regulating of various biological pathways, indicating promising potential for application development in fields such as oxidative energy production, protein metabolism regulation, fat metabolism regulation, immune modulation, oxidative stress alleviation, promotion of rumen microbial metabolism, and enhancement of production performance (Liu et al., 2023). Valine, an essential amino acid (EAA), is crucial for animal growth and development. Supplementing the diets of livestock and poultry with appropriate levels of Val has been shown to enhance immune and antioxidant capacities while improving production performance through the regulating of protein and glucose metabolism (Xia et al., 2023). Research conducted by Cai (2025) suggests that an optimal level of Val in diets can enhance the production performance of grass carp during the late growth stage. This enhancement is reflected in increased nutrient content, improved sensory quality, flavor, and health value of the muscle, as well as the promotion of muscle fiber proliferation. In diets based on corn and soybean meal for broiler chicks, Val is identified as the fourth limiting amino acid. Supplementing these diets with optimal levels of Val has been shown to promote skeletal and intestinal development, facilitates growth and nutrient absorption in the gut, and consequently improve broiler production performance (Allameh and Toghyani, 2019). Overall, Val significantly impacts meat quality and metabolism in animals, however, its effects on the meat quality and metabolism of Tibetan sheep remain unclear.

The rumen, the primary digestive organ in ruminant animals, play a crucial role by providing space and nutrients for microbial survival. Rumen microbes ferment plant feed into short-chain fatty acids (SCFAs), which are essential for regulating rumen physiological functions (Song et al., 2025). Additionally, the modulation of microbial communities and metabolism in Tibetan sheep has been associated with improved antioxidant status and cellulase activity in the rumen (Zhang et al., 2024). Rumen microorganisms play a crucial role in influencing the production performance and meat quality of ruminants (Zhang et al., 2022). BCAAs have been shown to impact rumen function and the composition of rumen microbiota in these animals. When the concentrate-to-roughage ratio was maintained at 2:8, the addition of 2 mmol/L Leucine, 1.33 mmol/L Isoleucine, and 0.67 mmol/L Valine significantly increased dry matter degradation rates. This suggests that at these concentrations, BCAAs facilitate the degradation of fibrous substances in the rumen, particularly under low-protein dietary conditions. As a result, BCAAs enhance the population of rumen fiber-degrading bacteria, thereby improving the digestion of fibrous materials in the rumen. A Leucine: Isoleucine: Valine ratio of 3:1:2 promoted protozoan growth and increased both bacterial and microbial protein (MCP) content. The administration of branched-chain amino acids (BCAAs) in a ratio of Valine: Leucine: Isoleucine = 1:1:1 has been shown to enhance gut health, bolster immunity, and subsequently improve growth performance in calves (Zhao et al., 2024). Nevertheless, there is a paucity of research concerning the impact of valine supplementation on rumen function and microbial activity in ruminants.

Drawing form previous studies (Ma et al., 2023; Jia et al., 2025), we propose the hypothesis that varying levels of valine supplementation will may lead to alterations in the rumen microbial community composition and muscle metabolites of Tibetan sheep, with a potential interaction between these factors. To investigate this, we conducted sensory evaluations and physicochemical analyses to evaluate the quality of Tibetan sheep meat, aiming to elucidate the effects of valine on meat edibility, sensory attributes, and nutritional value. Furthermore, we utilized advanced analytical techniques, including triple quadrupole liquid chromatography-mass spectrometry (QQQ LC–MS), gas chromatography-tandem mass spectrometry (GC–MS/MS), Vanquish LC ultra-high-performance liquid chromatography (UHPLC), and Orbitrap Exploris™ 480 mass spectrometer, to assess the influence of varying valine levels on the amino acid, fatty acid, and metabolite profiles in the *longissimus dorsi* muscle of Tibetan sheep. A KEGG enrichment analysis was conducted on differential metabolites to elucidate the impact of Valine on metabolic pathways within the muscle tissue of Tibetan sheep. Subsequently, 16S rDNA sequencing was utilized to assess the effects of varying Valine concentrations on the abundance and composition of rumen microbiota in these sheep. Concurrently, correlations were examined between differentially abundant rumen microbes and muscle metabolites, as well as the relationship between these factors and meat quality. The objective was to uncover the intrinsic relationship between varying Valine levels (specifically, 0.1% and 0.15% treatment groups) and the resulting variations in the quality of Tibetan sheep meat. This study endeavors to establish a theoretical foundation for the development of standardized precision nutrition management protocols for Tibetan sheep, thereby contributing to the advancement of the livestock industry in Qinghai.

## 2 Materials and methods

The research was conducted at the Tibetan Sheep Breeding Center located in Gonghe County, Qinghai Province, with approval from the Animal Ethics Committee of Qinghai University (Approval No. QUA-2022-1972, 2022.07.02).

### 2.1 Experimental animals and sample collection

Ninety healthy Tibetan sheep lambs, each approximately 2 months old and exhibiting similar body conditions, were randomly allocated into three groups of 30 for dietary experimentation. After a 14-day acclimatization period, a formal 120-day feeding trial was initiated. Group K functioned as the control group, receiving only the basal diet. In contrast, experimental groups V1 and V2 were supplemented with 0.1 and 0.15% Val concentrate (Valine: using corn and inorganic salts as raw materials, the glucose in corn hydrolysate serves as the carbon source, while inorganic salts provide essential nutrients such as nitrogen, phosphorus, and potassium. Through metabolic regulation—such as relieving metabolic feedback inhibition—valine is synthesized. Content: 98.0–101.0%, product standard code: GB 7300 104, Ningxia Yipin Biotechnology Co., Ltd., Yinchuan, China), supplement each sheep with 0.6 kg of concentrate basal daily. Respectively, in addition to the basal diet. The basal diet for all groups comprised concentrate supplements, silage oat hay, and green oat hay, with specific proportions of the concentrate supplement detailed in Table 1. All Tibetan sheep were vaccinated against Tetralogy of Fallot, Peste des Petits Ruminants (PPR), Foot-and-Mouth Disease (FMD), and Sheep Pox. Furthermore, regular administrations of Ivermectin and Abamectin were conducted to prevent and control both internal and external parasitic infections.

**Table 1:** Dietary concentrate supplementation.

Raw material composition/%	K	V1	V2
Maize	52	51.9	51.85
Wheat	10	10	10
Palm kernel meal	15	15	15
Soybean meal	5	5	5
Rapeseed meal	14	14	14
Common salt	1	1	1
Stone powder	1	1	1
Baking soda	1	1	1
1% Premix (Lamb)	0.6	0.6	0.6
4% Concentrate (No. 3)	0.4	0.4	0.4
Valine	0	0.1	0.15
Total	100	100	100

The 4% concentrate used does not contain Val. K: basic diet without Val; V1: diet containing 0.10% Val; V2: diet containing 0.15% Val.

At the conclusion of the trial, six animals from each group were randomly selected. Following a 12-h fasting period, these animals were transported to a humane slaughter facility. In compliance with animal welfare protocols, all animals were slaughtered humanely. Prior to slaughter, approximately 20 mL of blood was collected via venipuncture into vacuum tubes. The blood was allowed to clot at room temperature for 4 h and subsequently centrifuged at 3000 rpm at 4 °C for 10 min to obtain the supernatant. Post-mortem, the *longissimus dorsi* muscle, located between the 9th and 11th ribs on the back of the Tibetan sheep, and rumen fluid were collected for further analysis. Slaughtering and sampling were conducted by trained personnel in accordance with standardized protocols. The collected *longissimus dorsi* samples were rinsed with sterile saline and placed in 2 mL cryogenic tubes. All samples were initially stored in liquid nitrogen tanks and, upon return to the laboratory, were transferred to a − 80 °C freezer for future use.

### 2.2 Determination of quality analysis of Tibetan sheep

#### 2.2.1 Determination of growth performance and slaughter quality

Animals were fasted for 24 h prior to slaughter. Body diagonal length, height at withers, and chest circumference were measure using a flexible tape measure. Live body weight was recorded before slaughter and carcass weight was measured post-slaughter to calculate the dressing percentage. Rib meat thickness, abdominal wall thickness, and backfat thickness were assessed using a vernier caliper. Trace the eye muscle outline using tracing paper and calculate eye muscle area (Ma et al., 2023).

#### 2.2.2 Determination of the sensory quality of the *longissimus dorsi* muscle

Utilizing a meat sensory evaluation methodology, a panel comprising 10 individuals (5 males and 5 females) trained in sensory assessment (Miller, 2023) performed a sensory analysis of Tibetan mutton, assessing its texture, elasticity, flavor, palatability, and overall quality (Ma et al., 2023). The evaluators were situated in a controlled environment, with each participant occupying an individual space where environmental conditions were maintained as consistently as possible. Pure water and crackers were provided during testing to act as palate cleansers. In each evaluation session, each participant randomly assessed three sets of muscle samples. The evaluators rated the meat’s texture, elasticity, flavor, palatability, and overall quality according to the criteria detailed in Supplementary Table S1.

#### 2.2.3 Determination of the edible quality of the *longissimus dorsi* muscle

Edible quality was evaluated following the methodology described by Jia et al. (2025). Following calibration of the pH probe (pHS-S3C, Shanghai Leizhi Instrument Factory, Shanghai, China) to pH values of 4.0 and 6.86, the probe was inserted into the center of the meat sample to measure pH. The colorimetric parameters, specifically a* (redness), b* (yellowness), and L* (lightness), of the *longissimus dorsi* muscle were quantified utilizing an auto-calibrating colorimeter (ADCI-60-C, Beijing ChenTec Instrument Technology Co., Ltd., Beijing, China). To assess thawing loss, samples were thawed at 4 °C for 12 h. Cooking loss was determined by immersing the samples in a constant-temperature water bath (HH-6, Changzhou Jintan Youlian Instrument Research Institute, Jiangsu, China) maintained at 80 °C for a duration of 30 min. Post-thaw, samples were sectioned into blocks measuring 2 × 1 × 1 cm, oriented along the muscle fiber direction. Shear force measurements were conducted perpendicular to the muscle fibers using a tenderness tester (MAQC-12, Nanjing Xiyi Instrument Equipment Co., Ltd., Nanjing, China). A circular sampler with a 5 cm diameter was employed to extract samples approximately 1 cm thick from the center of thawed specimens. The water-holding capacity was evaluated using a water-holding capacity tester (MAEC-18, Nanjing Mingao Instrument Equipment Co., Ltd., Nanjing, China) under a pressure of 350 N. Finally, the meat samples were analyzed for textural properties, including hardness, elasticity, viscosity, adhesiveness, and chewiness using a texture analyzer (CT3-10 K, Brookfield, Massachusetts, United States).

#### 2.2.4 Determination of the nutritional quality of the *longissimus dorsi* muscle

To assess the nutritional quality of meat samples, the moisture, protein, and fat content should be determined in accordance with established meat testing standards. For moisture content analysis, place the sample in an electric forced-air drying oven (model DHG-9070A, Shanghai Yiheng Technology Co., Ltd., Shanghai, China) set at 105 ± 2 °C and heat until a constant mass is achieved. Protein content is quantified using an automatic Kjeldahl nitrogen analyzer (model K9840, Shandong Haineng Scientific Instrument Co., Ltd., Shandong, China), while fat content is measured with a total fat analyzer (model MF-105, Shanghai Xianjian Instrument Co., Ltd., Shanghai, China).

### 2.3 Determination of targeted metabolomics analysis of the *longissimus dorsi* muscle

#### 2.3.1 Determination of AA composition

Prior to analysis, slowly thaw the sample at 4 °C, then take an appropriate amount and combine it with a mixture of isotope internal standard and a pre-chilled methanol/acetonitrile/water solution (2:2:1, v/v/v). This mixture is homogenized using a vortex mixer (model QT-1, Shanghai Qite Analytical Instrument Co., Ltd., Shanghai, China) and incubated at −20 °C for 1 h to facilitate protein precipitation. Subsequent centrifugation at 14,000 rcf and 4 °C for 20 min yields a supernatant, which is then vacuum-dried for further analysis. The samples were initially separated utilizing a 1290 Infinity UPLC system (Agilent Technologies, City of Santa Clara, United States) and subsequently analyzed via mass spectrometry in Multiple reaction Monitoring (MRM) mode with a 6,500+ QTRAP mass spectrometer (SCIEX).

#### 2.3.2 Determination of FA composition

Following a gradual thawing process at 4 °C, 5 mL of a dichloromethane-methanol solution (2:1 v/v) was added to the sample, which was then vortex to ensure through mixing. Subsequently, 2 mL of gold-labeled water was introduced for washing purposes, and the lower layer of the solution was collected and evaporated to dryness under a nitrogen stream. Thereafter, 2 mL of n-hexane was added, along with the incorporation of an internal standard, followed by methylation for 30 min. An additional 2 mL of gold-labeled water was then added, and 1,000 μL of the supernatant was aspirated and evaporated to dryness under nitrogen. The residue was redissolved in n-hexane, and the supernatant was transferred to a sample vial for separation using a capillary column (Agilent DB-23, 60 m × 250 μm × 0.15 μm) within a gas chromatography system. Finally, mass spectrometry analysis was conducted using a 5977B MSD mass spectrometer (Agilent Technologies, City of Santa Clara, United States).

### 2.4 Determination of untargeted metabolomics analysis of the *longissimus dorsi* muscle

The samples were separated utilizing a Vanquish LC ultra-high-performance liquid chromatography (UHPLC) system equipped with a HILIC column (Thermo Scientific), and subsequently analyzed via mass spectrometry using an Orbitrap Exploris™ 480 mass spectrometer (Thermo Scientific) in both electrospray ionization (ESI) positive and negative ion modes.

### 2.5 Determination of AA composition in serum

The amino acid contents in the serum of Tibetan sheep were determined using the methods described in Section 2.3.1.

### 2.6 Determination of rumen metabolites and microorganisms

#### 2.6.1 Determination of AA composition in rumen fluid sample

The amino acid contents in the rumen fluid of Tibetan sheep were determined using the methods described in Section 2.3.1.

#### 2.6.2 The pH of rumen fluid

Post-slaughter, rumen fluid from Tibetan sheep was collected and filtered through four layers of coarse gauze with a mesh size of 250 mm. The filtered fluid was then transferred into 15 mL test tubes. The pH value was measured using a glass electrode connected to a portable pH meter (model PHS-3C, Shanghai Leizhi Instrument Factory, Shanghai, China), with each sample measured three times to obtain an average value.

#### 2.6.3 SCFAs concentration in rumen fluid

Following slow thawing at 4 °C an appropriate volume of the sample was resuspended in 50 μL of a 20% phosphoric acid buffer. A diethyl ether solution containing a 500 μM internal standard was added, and the mixture was thoroughly homogenized. The sample was then centrifuged at 14,000 g for 20 min at 4 °C, and the supernatant was transferred to a sample vial. Separation was conducted using a gas chromatography system equipped with an Agilent DB-FFAP capillary column (30 m × 250 μm × 0.25 μm), followed by an Agilent 5977B MSD mass spectrometer.

#### 2.6.4 Determination of rumen microbial composition

Following the natural thawing of rumen fluid at 4 °C, its purity and concentration were evaluated in accordance with the methodology outlined by Zhang et al. (2023). Genomic DNA was extracted from the samples utilizing the OMEGA Mag-bind soil DNA Kit, and subsequent measurements of DNA purity and concentration were conducted. PCR amplification of the V3-V4 variable region was executed using barcoded specific primers and a high-fidelity DNA polymerase, tailored to the selected sequencing region. The PCR products were visualized through 2% agarose gel electrophoresis, and target fragments were isolated from the gel employing the Quant-iT PicoGreen dsDNA Assay Kit. Preliminary electrophoresis quantification results informed the quantification of PCR-amplified recovered products, which was performed using a Microplate Reader (BioTek, FLx800) fluorescence quantification system. Samples were then combined in appropriate ratios to meet the sequencing volume requirements for each sample. Library preparation was conducted using Illumina’s TruSeq Nano DNA LT Library Prep Kit. The constructed libraries underwent rigorous quality control using the Agilent Bioanalyzer 2,100 and Promega QuantiFluor systems. Upon successful quality control, the libraries proceeded to sequenced.

### 2.7 Data processing and analysis

Differences between indicators of Tibetan sheep carcass, edible quality, and nutritional quality were conducted utilizing a one-way analysis of variance (ANOVA) in IBM SPSS Statistics 27 software. The results are presented as the mean ± standard error of the mean (X ± SEM). Differences between means were evaluated using Duncan’s multiple range test. Normality of continuous variables was evaluated using the Shapiro–Wilk test, while homogeneity of variance was assessed using Levene’s test. Results were determined statistically significance at *p* < 0.05. Bar charts were created using Origin Pro 2021, and orthogonal partial least squares discriminant analysis (OPLS-DA) models were developed using SIMCA 14.1. Pearson correlation coefficients were employed to analyze the correlations between muscle quality and metabolites, as well as between SCFAs and rumen microbiota (*p* < 0.05 indicates a significant correlation, and *p* < 0.01 indicates an extremely significant correlation). For metabolomics data analysis, the XCMS software platform was employed for metabolite identification and quantification, including peak alignment, retention time correction, and peak area extraction. Specifically, functional annotation of differential metabolites and their associated metabolic pathway analysis were performed using the Kyoto Encyclopedia of Genes and Genomes (KEGG) database (KEGG^1^). α, β diversity indices of rumen samples were calculated using QIIME 2.

## 3 Results

### 3.1 Effects of valine supplementation on the growth performance and carcass quality of Tibetan sheep

The slaughter characteristics of Tibetan sheep are indicative of carcass quality and serve as a crucial metric for evaluating their economic value and overall performance. These characteristics encompass parameters such as slaughter weight, carcass weight, meat yield rate, rib fat thickness, abdominal fat thickness, backfat thickness, eye muscle area, height, chest circumference, and body oblique length. As illustrated in Table 2, the initial weight, slaughter weight, and carcass weight of the V1 group were significantly higher than those observed in the K and V2 groups, whereas the body height of the Tibetan sheep was significantly lower compared to the K and V2 groups (*p* < 0.05). This indicates that valine supplementation may have no significant effect on weight changes in Tibetan sheep. With increasing levels of Val, there was a significant enhancement in meat yield rate and rib fat thickness (*p* < 0.001), with the V2 group exhibiting a 3.61% increase in meat yield rate relative to the K group. Suggesting that valine supplementation may have enhanced the production performance of Tibetan sheep. The abdominal fat thickness in the V2 group was significantly greater than that in the K group. Additionally, the backfat thickness in the K group was significantly lower than that in the V1 and V2 groups, while its chest circumference was significantly greater than that of the V1 and V2 groups (*p* < 0.05). This indicates that supplementing feed with Val affects the slaughter traits of Tibetan sheep.

**Table 2:** Effect of valine supplementation on the growth performance and carcass traits of Tibetan sheep.

Butchery trait	Groups			*p*-value
	K	V1	V2	
Initial weight (kg)	16.73 ± 0.68^b^	17.77 ± 0.33^a^	16.42 ± 0.45^b^	0.040
Slaughter weight (kg)	35.08 ± 0.34^b^	37.03 ± 0.55^a^	35.65 ± 0.15^b^	0.002
Carcass weight (kg)	16.43 ± 0.21^b^	17.40 ± 0.17^a^	16.27 ± 0.31^b^	0.002
Meat yield rate (%)	52.65 ± 0.18^c^	53.83 ± 0.07^b^	54.55 ± 0.16^a^	<0.001
Average daily weight gain (kg)	0.22 ± 0.01	0.21 ± 0.01	0.22 ± 0.00	0.296
Rib fat thickness (mm)	25.49 ± 0.08^c^	26.44 ± 0.50^b^	29.09 ± 0.57^a^	<0.001
Abdominal fat thickness (mm)	21.42 ± 0.64^b^	23.39 ± 0.45^ab^	25.02 ± 1.87^a^	0.026
Backfat thickness (mm)	19.74 ± 0.70^b^	21.84 ± 0.67^a^	22.00 ± 1.41^a^	0.055
Eye muscle area (cm^2^)	22.67 ± 0.58	22.67 ± 1.53	22.00 ± 1.00	0.709
Height (cm)	65.30 ± 1.00^a^	60.53 ± 0.70^b^	64.50 ± 2.44^a^	0.021
Chest circumference (cm)	85.27 ± 1.05^a^	79.60 ± 0.53^b^	80.60 ± 1.85^b^	0.003
Oblique length (cm)	72.80 ± 0.61	71.47 ± 1.56	71.77 ± 3.54	0.760

The same lowercase letters or no letters indicate no significant difference (*P* > 0.05), and different lowercase letters indicate significant difference (*P* < 0.05).

### 3.2 Effects of valine supplementation on the edible quality of Tibetan sheep

The edible quality of meat is primarily determined by factors such as color, texture, tenderness, and water-holding capacity. As illustrated in Table 3, the pH of Tibetan mutton in Group V2 at 45 min post-mortem was significantly lower than that observed in Groups K and V1 (*p* < 0.05). Following a 24-h period of acid drainage, the pH values of Groups V1 and V2 were significantly elevated compared to Group K (*p <* 0.05). Notably, Group K exhibited a higher initial pH value of 7.07 at 45 min, which subsequently declined more rapidly than in the other groups, resulting in a lower pH after 24 h. Conversely, Group V2 demonstrated the slowest rate of pH decline. In terms of color metrics, the L* and a* values for Group V2 were significantly lower than those for Groups K and V1, whereas the b* values for Groups V1 and V2 were significantly lower than those for Group K (*p* < 0.05). Regarding water-holding capacity, Group V2 experienced a significantly lower cooking loss rate compared to Group K. Additionally, as the levels of Val increased, the tenderness of Tibetan mutton improved. Both V1 and V2 groups exhibited significantly lower shear force values than the K group, with Group V2 demonstrating a 26.93% reduction in shear force compared to Group K. The Water-holding capacity and elasticity of the sample exhibited a significant increase, whereas hardness and chewability showed a significant decrease (*p* < 0.05). Consequently, the Tibetan mutton from the V2 group demonstrated superior edible quality relative to the other two groups.

**Table 3:** Effect of valine supplementation on the edible quality of Tibetan sheep.

Edible quality	Groups			*p*-value
	K	V1	V2	
pH_45 min_	7.07 ± 0.06^a^	7.02 ± 0.18^a^	6.77 ± 0.02^b^	0.030
pH_24 h_	5.77 ± 0.09^b^	6.11 ± 0.07^a^	6.09 ± 0.03^a^	0.001
Color (45 min)				
L*	45.83 ± 0.74^a^	44.08 ± 1.66^a^	35.32 ± 0.51^b^	<0.001
a*	36.23 ± 2.75^a^	34.64 ± 2.90^a^	27.25 ± 4.19^b^	0.035
b*	43.57 ± 0.60^a^	28.32 ± 6.28^b^	24.88 ± 0.35^b^	0.002
Thawing loss (%)	4.94 ± 0.55	3.70 ± 1.02	4.96 ± 1.46	0.328
Cooking loss (%)	32.19 ± 0.72^a^	30.77 ± 0.97^ab^	29.74 ± 0.40^b^	0.018
Shear force (N)	27.55 ± 2.51^a^	22.50 ± 1.69^b^	20.13 ± 0.39^b^	0.006
Water-holding capacity (%)	16.62 ± 0.55^c^	19.61 ± 0.78^b^	23.35 ± 1.79^a^	0.001
Texture				
Hardness (g)	869.33 ± 70.09^a^	218.06 ± 23.06^c^	366.72 ± 8.42^b^	<0.001
Elasticity (mm)	3.31 ± 0.04^b^	3.58 ± 0.02^a^	3.69 ± 0.12^a^	0.002
Chewability (mJ)	13.84 ± 2.63^a^	4.22 ± 0.47^b^	6.20 ± 0.70^b^	<0.001
Viscosity (mJ)	1.18 ± 0.30	0.96 ± 0.13	0.96 ± 0.02	0.323
Cohesion (g)	0.62 ± 0.03^a^	0.60 ± 0.02^a^	0.54 ± 0.02^b^	0.021

The same lowercase letters or no letters indicate no significant difference (*P* > 0.05), and different lowercase letters indicate significant difference (*P* < 0.05).

### 3.3 Effects of valine supplementation on the sensory quality of Tibetan sheep

**Figure 1:**
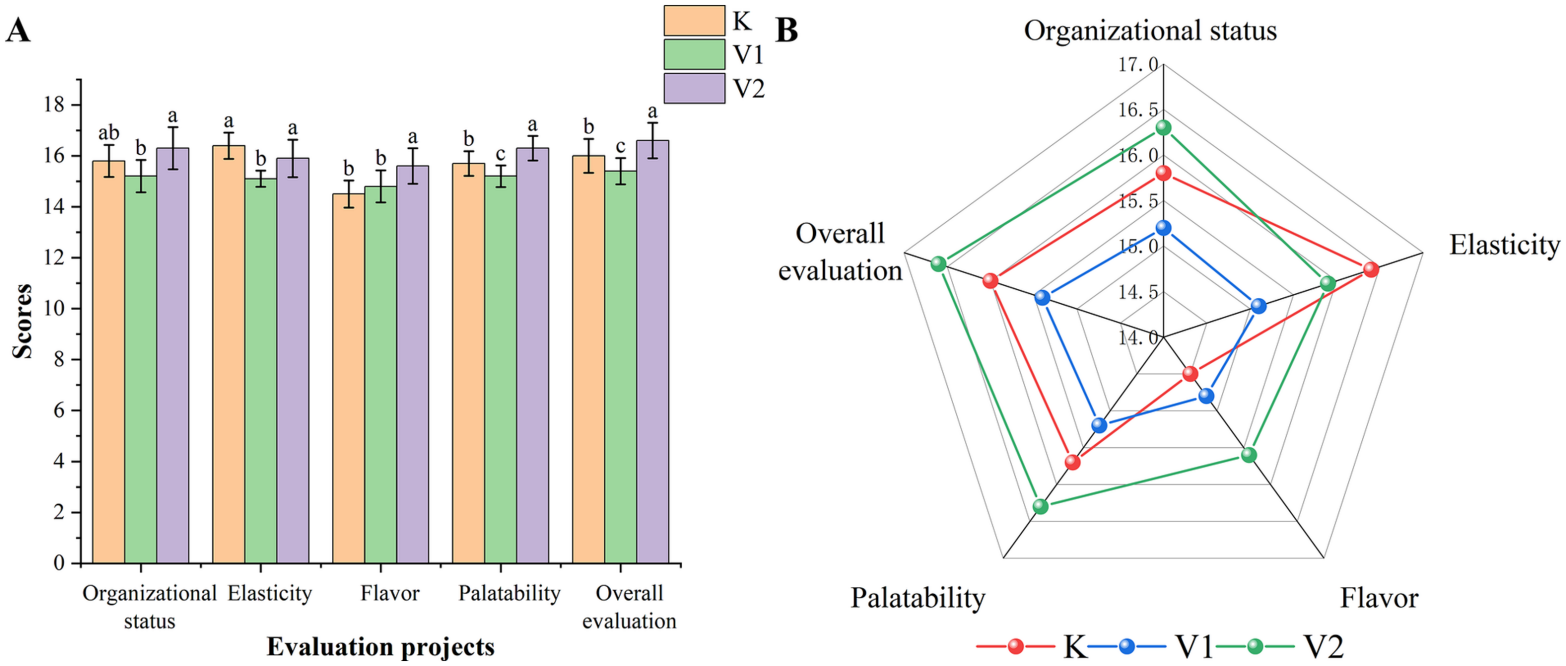
Sensory score histograms and radar charts. **(A)** sensory score histograms. **(B)** sensory score radar charts.

As illustrated in Figure 1A, the incorporation of varying levels of Val into the diet had a significant impact on the organizational status, elasticity, flavor, palatability, and overall evaluation of Tibetan sheep meat. Specifically, Group V2 exhibited a markedly superior organizational status compared to Group V1, while both Groups K and V2 demonstrated significantly enhanced elasticity relative to Group V1. The addition of different concentrations of Val improved the flavor of the meat compared to Group K, with Group V2 showing a notably greater effect on palatability and overall evaluation (*p* < 0.05). Figure 1B further indicates that Group V2 surpassed Group K in terms of flavor, palatability, and overall evaluation.

### 3.4 Effects of valine supplementation on the nutritional quality of Tibetan sheep

The nutritional quality of meat is conventionally assessed by analyzing crude fat, crude protein, and moisture content. As presented in Table 4, the addition of Val to the feed influenced the moisture content of Tibetan sheep meat, with the moisture content in Group V1 being significantly lower than that in Group K (*p* < 0.05). Both crude protein and crude fat contents increased in Groups V1 and V2, with the crude protein content in Group V2 being significantly higher than that in both Groups K and V1. The findings suggest that a diet enriched with Val facilitates the effective deposition of protein and fat in the muscle tissue of Tibetan sheep.

**Table 4:** Effect of valine supplementation on the nutritional quality of Tibetan sheep.

Nutritional quality	Groups			*p*-value
	K	V1	V2	
Moisture (%)	71.67 ± 1.36^a^	67.90 ± 1.49^b^	70.95 ± 1.88^ab^	0.059
Crude fat (g/100 g)	1.60 ± 0.44	1.87 ± 0.38	1.80 ± 0.98	0.878
Crude protein (g/100 g)	21.39 ± 0.82^b^	21.40 ± 0.80^b^	22.95 ± 0.55^a^	0.065

The same lowercase letters or no letters indicate no significant difference (*P* > 0.05), and different lowercase letters indicate significant difference (*P* < 0.05).

### 3.5 Effects of valine supplementation on AA content of Tibetan sheep

As presented in Table 5, Val supplementation in the diet influences the AA composition of the *longissimus dorsi* muscle in these sheep. With increasing levels of Val, there was a significant increase (*p* < 0.05) in the concentrations of aminoadipic acid and glutamine in the muscle tissue. The K group demonstrated significantly lower levels of phenylalanine, methionine, and EAAs in muscle tissue compared to the other two groups, whereas the levels of cysteine and ornithine were significantly higher (*p* < 0.05). In the V1 group, the concentrations of creatine, Ile, and Leu in muscle tissue were significantly higher than those in the K group. Additionally, the asparagine content was significantly elevated compared to both the K and V2 groups, while the choline content was significantly reduced (*p* < 0.05). In the V2 group, the levels of alanine and non-essential amino acids (NEAAs) in muscle tissue were significantly higher than those in the K group, but significantly lower than those in the V1 group (*p* < 0.05). Furthermore, the aspartate content was significantly higher than in the K group, whereas the glutamate content was significantly lower. The taurine concentration was significantly elevated compared to the other two groups (*p* < 0.05). In contrast, the Val feed did not exhibit a statistically significant difference in the total amino acid content within the *longissimus dorsi* muscle across the three groups of Tibetan sheep. Detailed measurement data for all amino acids are provided in Supplementary Table S2.

**Table 5:** Effect of valine supplementation on the AA content and composition of Tibetan sheep.

Amino acid composition	AA content (umol/kg)			*p*-value
	K	V1	V2	
Aminoadipic acid	25.64 ± 0.42^c^	38.21 ± 0.44^b^	69.32 ± 1.46^a^	<0.001
Ornithine	14.40 ± 0.17^a^	9.79 ± 0.39^b^	9.57 ± 0.10^b^	<0.001
Alanine	3679.35 ± 11.36^c^	4102.96 ± 40.06^a^	3844.92 ± 24.62^b^	0.001
Glutamine	221.99 ± 3.94^c^	392.71 ± 45.37^b^	492.36 ± 19.78^a^	0.006
Phenylalanine	112.59 ± 8.04^b^	160.40 ± 4.39^a^	153.22 ± 7.16^a^	0.011
Choline	17710.08 ± 150.60^a^	10872.15 ± 687.23^b^	15184.45 ± 1565.82^a^	0.014
Taurine	3046.56 ± 132.43^b^	2782.00 ± 225.71^b^	3807.60 ± 55.75^a^	0.014
Leucine	391.04 ± 25.07^b^	488.08 ± 0.35^a^	438.10 ± 18.46^ab^	0.028
Cysteine	0.74 ± 0.05^a^	0.27 ± 0.15^b^	0.16 ± 0.14^b^	0.035
Methionine	36.89 ± 12.82^b^	57.34 ± 2.20^a^	57.24 ± 4.78^a^	0.035
Asparagine	202.66 ± 4.20^b^	270.43 ± 22.04^a^	194.89 ± 18.32^b^	0.036
Glutamate	346.37 ± 14.47^a^	244.78 ± 44.31^ab^	184.93 ± 36.36^b^	0.039
Creatine	4151.26 ± 58.05^b^	4413.10 ± 2.42^a^	4305.21 ± 80.20^ab^	0.044
EAAs	2251.22 ± 259.76^b^	2759.93 ± 40.03^a^	2811.41 ± 26.36^a^	0.060
NEAAs	6136.41 ± 69.25^c^	7480.34 ± 229.97^a^	7002.33 ± 87.51^b^	0.006

The same lowercase letters or no letters indicate no significant difference (*P* > 0.05), and different lowercase letters indicate significant difference (*P* < 0.05). TAAs, total amino acids; EAAs, essential amino acids; NEAAs: non- essential amino acids.

### 3.6 Effects of valine supplementation on the FA content in the *longissimus dorsi* muscle

Table 6 demonstrates that the fatty acid (FA) composition in Tibetan sheep meat influenced by varying levels of Val-supplemented feed. In comparison to the control group (K), the V1 group exhibited significantly lower concentrations of C6:0, C10:0, and C23:0 (*p* < 0.05). Both V1 and V2 groups showed significantly reduced levels of C21:0, C22:0, C24:0, C15:1n5 cis, C18:2 n-6,9 all-cis, C18:2 n-6,9,12 all-cis, and C20:3 n-3,6,9 all-cis compared to the K group, whereas the docosapentaenoic acid (DPA) content was significantly elevated (*p* < 0.05). The total n-6 and total polyunsaturated fatty acid (PUFA) content in the K and V2 groups were significantly greater than in the V1 group (*p* < 0.05). Additionally, the total *n*−3 content in the V2 group was significantly higher than that in the K and V1 groups. No statistically significant differences were observed in the total saturated fatty acid (SFA) and total monounsaturated fatty acid (MUFA) levels among the three groups of Tibetan sheep *longissimus dorsi* muscle.

**Table 6:** Effects of valine supplementation on the FA content and composition of Tibetan sheep.

Fatty acid composition	FA content (ug/g)			*p*-value
	K	V1	V2	
C6:0	0.05 ± 0.01^a^	0.04 ± 0.00^b^	0.05 ± 0.00^ab^	0.057
C8:0	0.38 ± 0.08	0.29 ± 0.00	0.36 ± 0.01	0.299
C10:0	5.66 ± 0.57^a^	3.19 ± 0.85^b^	4.37 ± 0.40^ab^	0.067
C11:0	0.12 ± 0.03	0.09 ± 0.01	0.13 ± 0.04	0.452
C12:0	7.38 ± 3.29	7.32 ± 2.06	10.28 ± 2.83	0.547
C13:0	0.30 ± 0.11	0.24 ± 0.08	0.40 ± 0.16	0.531
C14:0	135.31 ± 77.23	153.96 ± 17.42	216.94 ± 82.73	0.514
C15:0	11.24 ± 4.42	10.14 ± 1.38	14.39 ± 4.87	0.583
C16:0	1614.21 ± 492.20	1903.19 ± 94.98	2124.50 ± 264.21	0.409
C17:0	38.37 ± 11.09	37.09 ± 3.93	48.41 ± 12.82	0.540
C18:0	1161.44 ± 145.75	1305.34 ± 40.84	1395.63 ± 73.67	0.195
C20:0	4.87 ± 1.32	5.37 ± 0.52	5.22 ± 0.27	0.836
C21:0	0.72 ± 0.02^a^	0.54 ± 0.00^b^	0.55 ± 0.01^b^	0.002
C22:0	0.64 ± 0.05^a^	0.38 ± 0.07^b^	0.41 ± 0.06^b^	0.038
C23:0	0.14 ± 0.01^a^	0.10 ± 0.02^b^	0.12 ± 0.01^ab^	0.076
C24:0	0.25 ± 0.02^a^	0.14 ± 0.03^b^	0.15 ± 0.02^b^	0.025
C14:1 N-5	4.60 ± 3.20	5.64 ± 0.95	7.03 ± 2.19	0.622
C15:1n5 cis	0.18 ± 0.00^a^	0.03 ± 0.01^b^	0.04 ± 0.02^b^	0.003
C16:1 N-7 cis	151.11 ± 70.07	261.07 ± 44.41	209.87 ± 16.94	0.227
C17:1 N-7 cis	36.32 ± 10.33	43.94 ± 4.04	44.45 ± 2.82	0.479
C18:1 N-9	2972.38 ± 571.93	3548.64 ± 295.15	3737.86 ± 173.89	0.264
C18:2 N-6,9 all-cis	763.15 ± 4.06^a^	594.26 ± 22.03^b^	627.47 ± 69.26^b^	0.053
C18:1 N-9 T	19.51 ± 0.89	28.26 ± 3.80	23.69 ± 3.18	0.124
C18:2 N-6,9 all-trans	9.45 ± 2.11	9.93 ± 0.42	10.54 ± 0.48	0.719
C18:3 N-3,6,9 all-cis	51.80 ± 7.88	58.30 ± 3.46	50.43 ± 3.32	0.404
C18:2 N-6,9,12 all-cis	8.84 ± 0.14^a^	7.78 ± 0.35^b^	8.09 ± 0.02^b^	0.036
C20:1 N-9 cis	16.12 ± 2.53	13.32 ± 0.55	11.68 ± 0.86	1.14
C20:2 N-6,9 all-cis	40.02 ± 5.75	44.96 ± 2.10	43.42 ± 1.69	0.479
C20:3 N-6,9,12 all-cis	21.93 ± 1.64	25.29 ± 2.53	23.72 ± 0.38	0.301
C20:3 N-3,6,9 all-cis	4.16 ± 0.14^a^	3.26 ± 0.21^b^	3.50 ± 0.12^b^	0.023
EPA	31.06 ± 0.02	34.51 ± 1.39	35.70 ± 3.91	0.278
C22:1 N-9	0.92 ± 0.07	1.21 ± 0.03	0.83 ± 0.23	0.144
C22:4 N-6,9,12,15 cis	15.05 ± 2.75	14.61 ± 1.17	13.98 ± 0.08	0.833
C24:1 N-9 cis	1.64 ± 0.14	1.72 ± 0.23	1.72 ± 0.12	0.848
DPA	61.86 ± 0.18^b^	75.97 ± 2.13^a^	73.07 ± 1.77^a^	0.006
ARA	363.15 ± 38.74	300.03 ± 21.08	350.59 ± 7.34	0.172
DHA	10.44 ± 0.19	10.84 ± 0.38	10.91 ± 0.53	0.520
Total SFA	2980.04 ± 737.30	3427.57 ± 160.73	3821.93 ± 441.10	0.373
Total MUFA	3203.55 ± 658.18	3829.08 ± 359.18	4037.47 ± 198.08	0.296
Total PUFA	1393.50 ± 4.72^a^	1176.94 ± 21.86^b^	1404.69 ± 96.82^a^	0.047
Total N3	178.79 ± 2.93^b^	177.81 ± 2.63^b^	193.90 ± 6.90^a^	0.065
Total N6	1221.12 ± 22.74^a^	993.20 ± 13.15^b^	1197.42 ± 85.45^a^	0.038

The same lowercase letters or no letters indicate no significant difference (*P* > 0.05), and different lowercase letters indicate significant difference (*P* < 0.05). EPA: 5Z,8Z,11Z,14Z,17Z-eicosapentaenoic acid; DPA: 7Z,10Z,13Z,16Z,19Z-docosapentaenoic acid and 4Z,7Z,10Z,13Z,16Z-docosapentaenoic acid; ARA: arachidonic acid; DHA: 4Z,7Z,10Z,13Z,16Z,19Z-docosahexaenoic acid; SFA: sum of saturated fatty acid; MUFA: sum of monounsaturated fatty acids; PUFA: sum of polyunsaturated fatty acids; Total N3: total omega-3 polyunsaturated fatty acids; Total N6: total omega-6 polyunsaturated fatty acids.

### 3.7 Untargeted metabolomics analysis of Tibetan sheep meat

#### 3.7.1 Quality control analysis

**Figure 2:**
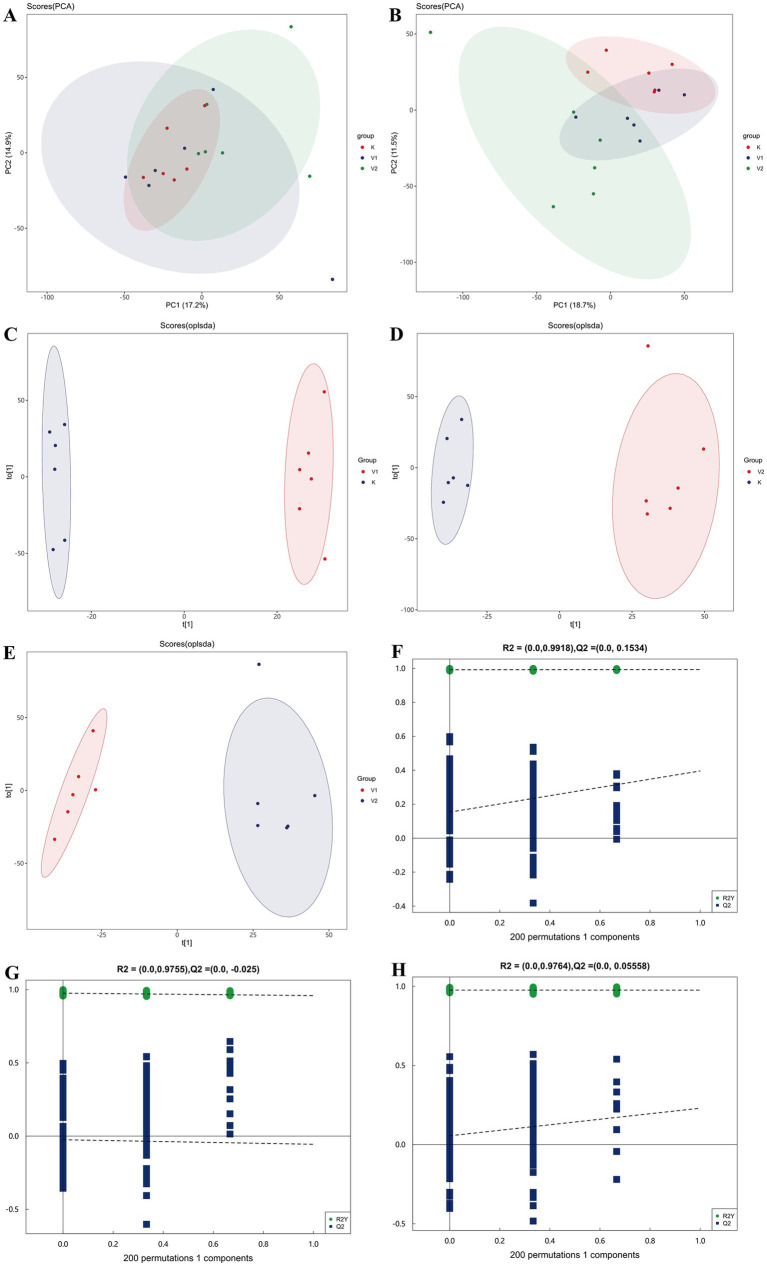
Plots of PCA scores for all samples in positive **(A)** and negative **(B)** ion modes; **(C–E)** OPLS-DA scores for overall samples in positive ion mode; **(F–H)** permutation test for OPLS-DA in positive ion mode.

To further explore the impact of supplemental valine on the *longissimus dorsi* muscle mass in Tibetan sheep, non-targeted metabolomic profiling was conducted using UHPLC-Orbitrap Exploris 480 MS in both positive and negative ionization modes. The experimental findings revealed significant overlap in the response intensity and retention time of each chromatographic peak, indicating minimal variation attributable to instrumental error throughout the study. As illustrated in Figures 2A,B, principal component analysis (PCA) scores were utilized to visualize differences among the three experimental groups. While no distinct separation was observed in the positive ion mode, more pronounced separation was evident in the negative ion mode. The R^2^X values for the positive and negative ionization modes were 0.506 and 0.513, respectively. Consequently, the negative ion mode was selected for further analysis to ensure more reliable data.

To enhance differentiation between groups, orthogonal partial least squares discriminant analysis (OPLS-DA) was applied, excluding quality control samples, to assess the metabolites in the samples. The R^2^Y value represents the cumulative variance explained by the model, with higher values indicating stronger explanatory power. Q^2^ represents the proportion of variance in the data that is predicted by the current model. As illustrated in Figures 2C–E, under the negative ion model, samples demonstrated clustering within groups and dispersion between groups. To mitigate the risk of overfitting during supervised modeling, permutation tests were conducted to validate the models (Figures 2F–H). As the number of permutation iterations increased, the R^2^ and Q^2^ values of the random model consistently declined, thereby confirming the absence of overfitting in the original models. These findings suggest that the metabolic separation between groups observed during the OPLS-DA analysis is statistically significant.

#### 3.7.2 Number of metabolites and chemical classification assignment

**Figure 3:**
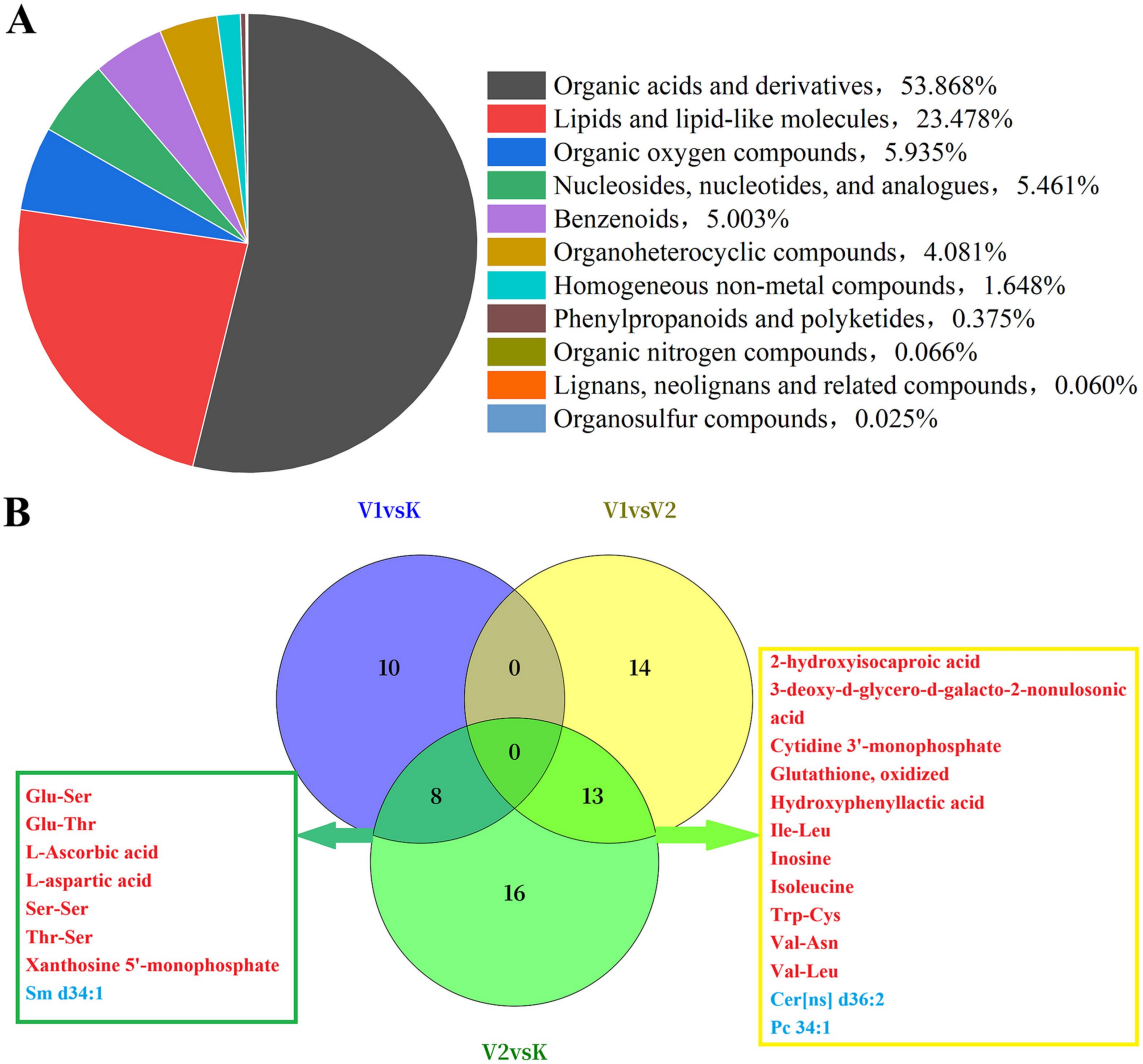
Chemical classification (parent category) assignment diagram **(A)** and Venn diagram **(B)** of muscle metabolites in Tibetan sheep under different VAL feeding levels.

In negative ion mode, 506 metabolites were identified through comprehensive differential metabolite analysis. By applying predefined criteria for differential metabolites (DFM) (*VIP* > 1 and *p* < 0.05), 102 key metabolites were identified across the three groups in negative ion mode. All identified metabolites were statistically categorized based on their chemical taxonomy. The proportion of metabolites in each category relative to their parent class is depicted in Figure 3A. Organic acids and their derivatives constituted the largest proportion at 53.868%, followed by lipids and lipid-like molecules, organic oxygen compounds, nucleosides, nucleotides, and analogs.

#### 3.7.3 Differential metabolite identification and analysis

Three sets of samples demonstrated metabolic variations at the superclass level, as depicted in the volcano plot (Supplementary Figures S1A–C). In the negative ion mode, when compared to K, there was an observed increase in organic acids and derivatives, as well as nucleosides, nucleotides, and analogs in V1 group, whereas organic oxygen compounds, phenylpropanoids and polyketides, and lipids and lipid-like molecules showed a decreasing trend (Supplementary Figure S1A); In the V2 group, benzenoids, organic heterocyclic compounds, organic oxygen compounds, organic acids and derivatives, and nucleosides, nucleotides, and analogs were upregulated, in contrast to the downregulation of lipids and lipid-like molecules (Supplementary Figure S1B). A comparative analysis between the V1 and V2 groups revealed that lipid and lipid-like molecules and organic heterocyclic compounds were upregulated in the V1 group (Supplementary Figure S1C), whereas benzenoids, organic acids and derivatives, organosulfur compounds, organic oxygen compounds, phenylpropanoids and polyketides, and nucleosides, nucleotides, and analogs were downregulated. To further elucidate the alterations in differential metabolites across the three sample groups, identification was conducted in negative ion mode. Pairwise comparisons between the V1 and K groups, V2 and K groups, and V1 and V2 groups revealed the identification of 18, 37, and 27 differentially expressed metabolites (DFMs), respectively, as illustrated in Figure 3B. Of these metabolites, eight were associated with both the V1 and V2 groups, with seven being up-regulated and one down-regulated in comparison to the K group. Additionally, 13 metabolites were specific to the other two groups.

#### 3.7.4 Metabolic pathway annotation and enrichment analysis

**Figure 4:**
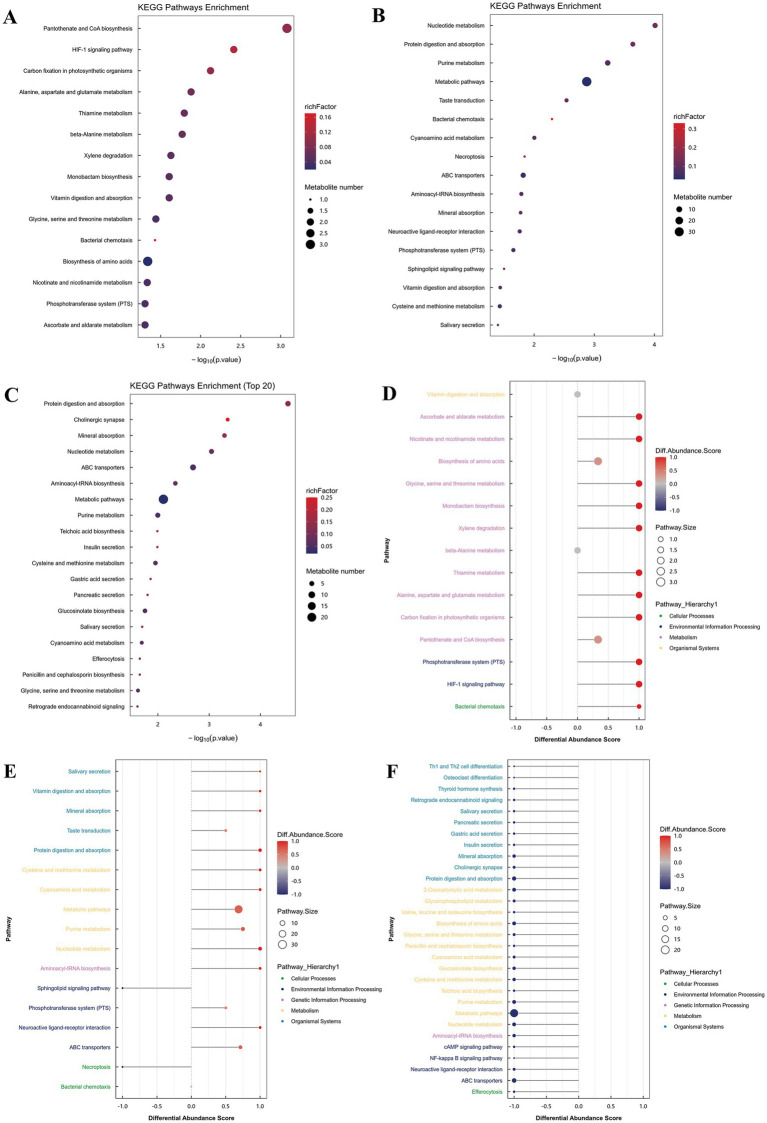
The 20 KEGG pathways with the highest enrichment in the comparison among the three groups **(A–C)**; differential enrichment score plots of different metabolic pathways in the longest dorsal muscle of Tibetan sheep **(D–F)**.

The KEGG pathway enrichment analysis, depicted in Figures 4A–C, identified the top 20 enriched pathways in Tibetan sheep meat subjected to three distinct levels of valine supplementation. In comparison to the K group, the V1 group exhibited pathways predominantly associated with amino acid biosynthesis, coenzyme and vitamin metabolism, energy metabolism, biosynthesis of other secondary metabolites, carbohydrate metabolism, phosphotransferase systems (PTS) (Figure 4A). Conversely, the V2 group was characterized by pathways related to metabolism, nucleotide metabolism, protein digestion and absorption, ABC transporters, mineral absorption, vitamin digestion and absorption, amino acid metabolism, and PTS (Figure 4B). In comparison to V2, the majority of V1 genes are implicated in processes such as metabolism, protein digestion and absorption, ABC transporters, mineral absorption, the biosynthesis of secondary metabolites, and amino acid metabolism (Figure 4C).

To further elucidate the impact of varying valine concentrations on metabolic pathways in Tibetan sheep, we conducted an analysis of the three groups using differential abundance scores (DA scores). As illustrated in Figures 4D–F, relative to the control group K, V1 demonstrated an upregulation in 11 metabolic pathways (DA scores > 0.5, *p* < 0.05). These pathways include the HIF-1 signaling pathway, carbon fixation in photosynthetic organisms, alanine, aspartate, and glutamate metabolism, thiamine metabolism, monobactam biosynthesis, glycine, serine and threonine metabolism, nicotinate and nicotinamide metabolism, ascorbate and aldarate metabolism, and the PTS (Figure 4D). Notably, the upregulated metabolites within these pathways comprised pyruvate, L-aspartic acid, L-ascorbic acid, and thiamine monophosphate (Supplementary Table S3).

In comparison to Group K, Group V2 exhibited upregulation in ten metabolic pathways (DA scores > 0.5, *p* < 0.05), encompassing nucleotide metabolism, protein digestion and absorption, purine metabolism, ABC transporters, vitamin digestion and absorption, and cysteine and methionine metabolism. Within these pathways, the upregulated metabolites included His-Ser, indole, inosine, capric acid, Ile, L-aspartic acid, L-ascorbic acid, inosine-5′-monophosphate, D-proline, DL-tryptophan, cysteine, guanosine 3′,5′-cyclic monophosphate, xanthine nucleoside 5′-monophosphate, oxidized glutathione, L-methionine, nicotinamide, tryptophan-cysteine, hypoxanthine, and xanthine nucleoside, among others (refer to Supplementary Table S3). Conversely, two pathways were downregulated, specifically those associated with necroptosis and sphingolipid signaling pathways (see Figure 4E). The downregulated metabolites included Cer(d18:1/18:1(9Z)) and N-(octadecanoyl) sphing-4-enine-1-phosphocholine (refer to Supplementary Table S3).

In comparison to Group V2, Group V1 demonstrated downregulated in 30 pathways (DA scores > 0.5, *p* < 0.05). The study identified a downregulation in several metabolic pathways, including these involved in protein digestion and absorption, mineral absorption, nucleotide metabolism, ABC transporters, aminoacyl-tRNA synthesis, general metabolic pathways, purine metabolism, cysteine and methionine metabolism, glucuronide biosynthesis, glycine, serine, and threonine metabolism, amino acid biosynthesis, glycerophospholipid metabolism, and 2-oxoglutarate metabolism, as illustrated in Figure 4F. Within these critical metabolic pathways, specific metabolites such as isoleucine, indole, DL-tryptophan, L-methionine, L-valine, deoxyadenosine, inosine, tryptophan-cysteine, 2′-deoxyinosine, hypoxanthine, 5-aminoacetyltyrosine, choline, methionine sulfone, cysteine, S-methyl-5′-thioadenosine, 2-aminohexanedioic acid, 1-stearoyl-2-hydroxy-sn-glycerol-3-phosphocholine, acetylcholine, D-inositol-1,4,5-trisphosphate, oxidized glutathione, L-guanosine, N-acetylneuraminic acid, and N-acetylglucosamine were also found to be downregulated, as detailed in Supplementary Table S3.

Overall, amino acid metabolism exhibited significant upregulation in the V1 and V2 groups, whereas the K group demonstrated a downregulation relative to the other groups. This study demonstrates that varying concentrations of Val in the diet significantly influence amino acid metabolism, as well as protein digestion and absorption, in the *longissimus dorsi* muscle of Tibetan sheep. This finding further substantiates the substantial impact of Val supplementation on muscle metabolism in these animals.

### 3.8 Correlation analysis between meat quality and untargeted metabolites

**Figure 5:**
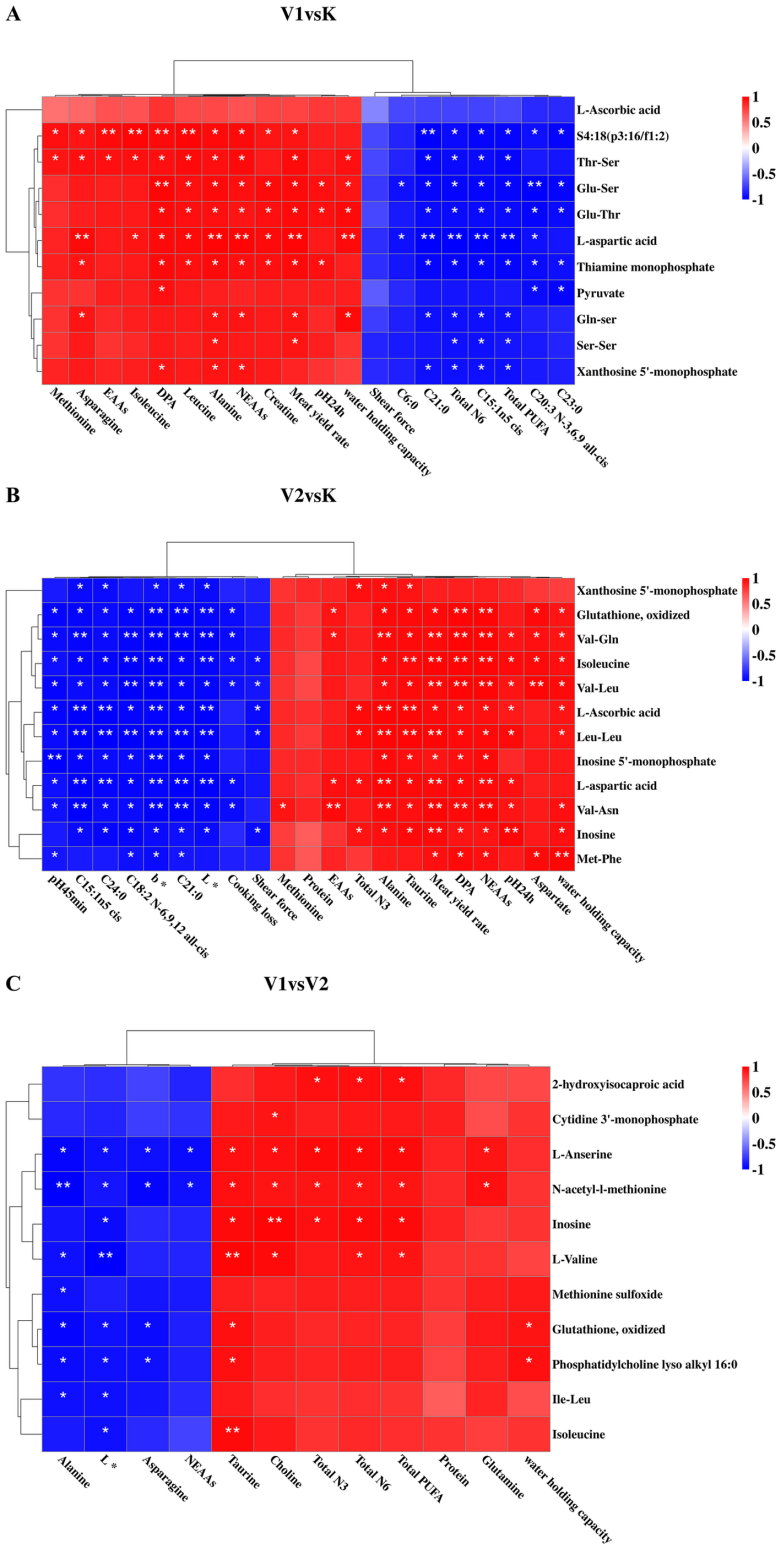
Clustered heat map of correlations between muscle metabolites and meat quality. **(A)** Clustering heat map showing the correlation between muscle metabolites and meat quality in V1vsK. **(B)** Clustering heat map showing the correlation between muscle metabolites and meat quality in V2vsK. **(C)** Clustering heat map showing the correlation between muscle metabolites and meat quality in V1vsV2. * indicates *p* < 0.01, ** indicates *p* < 0.001.

To elucidate the relationship between muscle metabolites and meat quality in Tibetan sheep subjected to different levels of Val-supplemented feed, correlation analyses were performed between phenotypic meat data and untargeted metabolomics results. As illustrated in Figure 5, robust correlations were identified between meat quality parameters and muscle metabolites across all experimental groups.

Specifically, Figure 5A presents a correlation clustering heatmap that delineates the associations between muscle metabolites and meat quality in the V1 and K groups of Tibetan sheep. Notably, pH_24h_ showed positive correlations with thiamine monophosphate, Glu-Thr and Glu-Ser. Additionally, water holding capacity exhibited positive correlations with Gln-Ser, L-ascorbic acid, Thr-Ser, Glu-Thr and Glu-Ser. Furthermore, the meat yield rate was positively correlated with Gln-Ser, L-ascorbic acid, thiamine monophosphate, Thr-Ser, Glu-Thr and Glu-Ser. The majority of fatty acids (FAs) that exhibited significant differences between groups were negatively correlated with differential metabolites in muscle tissue, whereas DPA demonstrated a significant positive correlation with these metabolites. Differential AAs generally showed positive correlations with metabolites. As depicted in Figure 5B, the correlation clustering heatmap elucidates the relationship between muscle metabolites and meat quality in Tibetan sheep from the V2 and K groups. Notably, negative correlations were identified between shear force, b* chroma value, and differential dipeptide metabolites, L-ascorbic acid, L-aspartic acid, inosine, and inosine 5′-monophosphate (IMP) in muscle tissue. Most FAs that were significantly different between groups exhibited negative correlations with differential muscle metabolites, while DPA maintained a significant positive correlation. Differential AAs were positively correlated with the majority of differential metabolites. Figure 5C depicts the association between muscle metabolites and meat quality in Tibetan sheep from V1 and V2 groups. Several significant correlations are discernible from the heatmap. For instance, the colorimetric L* values exhibit negative correlations with N-acetyl-L-methionine, isoleucine, L-valine, inosine and oxidized glutathione. Conversely, glutamine and polyunsaturated fatty acids demonstrate positive correlations with L-anserine and N-acetyl-l-methionine.

### 3.9 Effects of valine supplementation on the AA composition and concentration of Tibetan sheep serum

As indicated in Table 7, the supplementation of Val in the diet influences amino acid levels in the serum of Tibetan sheep. The serum concentrations of N-acetylneuraminic acid, creatine, creatinine, 4-acetamidobutanoic acid, 2-phenylglycine, methionine, pyroglutamic acid, pipecolic acid, and ornithine were significantly elevated compared to those in the V1 and V2 groups. Furthermore, the levels of 3-hydroxyhippuric acid, tyrosine, and 5-aminolevulinic acid were significantly higher than those in the V2 group. The choline content was significantly higher than in the V2 group but significantly lower than in the V1 group (*p* < 0.05).

**Table 7:** Effects of valine supplementation on the AA composition and concentration of Tibetan sheep serum.

Amino acid composition	AA content (ng/mL)			*p*-value
	K	V1	V2	
Choline	55174.26 ± 285.52^b^	60567.71 ± 285.64^a^	49878.52 ± 660.28^c^	<0.001
2-Phenylglycine	80.07 ± 1.77^a^	54.91 ± 0.92^b^	51.71 ± 0.54^b^	<0.001
Isoleucine	5297.02 ± 21.89^a^	5069.66 ± 35.59^b^	4744.20 ± 32.00^c^	<0.001
N-Methyl-aspartic acid	61.00 ± 0.70^a^	52.47 ± 0.40^b^	48.64 ± 0.10^c^	<0.001
Methionine	4460.69 ± 179.79^a^	3002.57 ± 63.47^b^	2904.76 ± 76.01^b^	0.002
N-Acetyltyrosine	168.27 ± 0.10^a^	161.51 ± 4.15^a^	131.89 ± 2.49^b^	0.002
Taurine	269.67 ± 12.26^c^	562.33 ± 42.10^a^	454.34 ± 1.53^b^	0.003
Valylalanine	2.19 ± 0.04^a^	2.11 ± 0.06^a^	1.79 ± 0.03^b^	0.006
2-Aminoisobutyric acid	7.77 ± 0.46^b^	7.95 ± 0.06^b^	10.25 ± 0.19^a^	0.006
α-aminobutyric acid	5.30 ± 0.44^b^	5.94 ± 0.08^b^	8.99 ± 0.66^a^	0.008
Pipecolic acid	51.80 ± 1.66^a^	39.77 ± 2.50^b^	38.07 ± 0.02^b^	0.008
N-Acetylneuraminic acid	52.64 ± 2.19^a^	37.69 ± 3.67^b^	33.38 ± 1.36^b^	0.010
Glycyl-glycine	5.43 ± 1.05^c^	10.68 ± 0.13^a^	8.31 ± 0.59^b^	0.011
N-Methylalanine	32.16 ± 0.39^a^	27.70 ± 3.08^a^	18.71 ± 0.63^b^	0.011
Glutamic acid	11357.51 ± 157.10^a^	10420.17 ± 284.39^b^	9414.94 ± 298.45^c^	0.011
Ornithine	2798.54 ± 509.60^a^	607.44 ± 31.30^b^	1044.97 ± 125.18^b^	0.011
Creatinine	4889.03 ± 495.23^a^	3130.92 ± 18.03^b^	2975.48 ± 7.30^b^	0.012
Aspartic acid	417.09 ± 57.43^c^	720.40 ± 144.89^b^	1074.79 ± 13.87^a^	0.012
Aminoadipic acid	193.38 ± 31.37^b^	296.53 ± 4.00^a^	322.92 ± 7.74^a^	0.012
Pyroglutamic acid	52.45 ± 0.04^a^	40.00 ± 3.69^b^	38.32 ± 0.78^b^	0.013
Betaine	400.93 ± 2.99^b^	432.29 ± 1.66^a^	418.99 ± 7.67^a^	0.017
Cystine	538.38 ± 65.09^a^	252.59 ± 17.60^b^	481.55 ± 42.40^a^	0.017
Serine	3874.10 ± 56.44^b^	5517.76 ± 367.22^a^	4834.73 ± 272.24^a^	0.019
Leucyl-glycine	8.25 ± 0.22^a^	8.61 ± 0.43^a^	5.64 ± 0.78^b^	0.020
2,3-Diaminopropionic acid	30.71 ± 1.33^b^	56.46 ± 6.96^a^	43.97 ± 1.93^ab^	0.021
S-Adenosylhomocysteine	72.45 ± 0.99^b^	76.20 ± 0.52^a^	75.20 ± 0.22^a^	0.022
5-Aminolevulinic acid	20.02 ± 3.23^a^	15.07 ± 0.33^ab^	9.69 ± 0.16^b^	0.027
Propionylglycine	62.71 ± 2.80^b^	99.63 ± 10.01^ab^	146.36 ± 25.02^a^	0.029
Carnosine	1763.41 ± 124.40^ab^	2049.26 ± 86.48^a^	1486.10 ± 100.51^b^	0.029
N-Acetylglutamic acid	30.63 ± 1.84^b^	36.42 ± 1.20^a^	30.64 ± 0.38^b^	0.032
Anserine	208.70 ± 16.00^ab^	228.44 ± 0.01^a^	182.18 ± 1.57^b^	0.035
Homoserine	3029.44 ± 266.42^ab^	2487.84 ± 272.50^b^	3757.29 ± 227.17^a^	0.036
N-Acetylaspartic acid	16.17 ± 1.04^b^	17.37 ± 2.81^b^	24.31 ± 0.96^a^	0.038
N6-Acetyllysine	33.94 ± 4.75^b^	41.91 ± 0.29^ab^	46.63 ± 0.25^a^	0.042
Threonine	4022.17 ± 391.83^ab^	3271.22 ± 394.24^b^	4884.41 ± 308.88^a^	0.049

The same lowercase letters or no letters indicate no significant difference (*P* > 0.05), and different lowercase letters indicate significant difference (*P* < 0.05).

In the V1 group, serum concentrations of γ-glutamyl-phenylalanine, N-isovaleroylglycine, and 2,3-diaminopropionic acid were significantly elevated compared to the K group. The content of N-acetylglutamic acid was significantly higher than that observed in both the K and V2 groups, while levels of anserine and carnosine were significantly elevated compared to the V2 group (*p* < 0.05). In the V2 group, serum levels of glycyl-glycine and taurine were significantly higher than those in the K group but significantly lower than those in the V1 group. Additionally, levels of phenylacetylglutamine, sarcosine, hydroxyproline, threonine, β-alanine, cis-4-hydroxy-D-proline, and homoserine were significantly elevated compared to the V1 group. Concentrations of proline, propionylglycine, γ-glutamylalanine, cystathionine, 2,6-diaminopimelic acid, and N6-acetyllysine were significantly higher than those in the K group, while levels of α-aminobutyric acid, 2-aminoisobutyric acid, and N-acetylaspartic acid were significantly elevated compared to both the K and V1 groups (*p* < 0.05). Furthermore, serum levels of valylalanine, leucylglycine, N-methylalanine, and N-acetyltyrosine in Tibetan sheep from the K and V1 groups were significantly higher than those in the V2 group (*p* < 0.05). In the serum of Tibetan sheep, cystine concentrations in Groups K and V2 were significantly elevated compared to Group V1 (*p* < 0.05). As the levels of valine supplementation increased, there was a significant decrease in serum isoleucine, N-methyl-D-aspartic acid, and glutamic acid concentrations, whereas aspartic acid concentrations increased significantly (*p* < 0.05). Furthermore, the concentrations of betaine, serine, S-adenosylhomocysteine, and aminoadipic acid in the serum of Tibetan sheep from Groups V1 and V2 were significantly higher than those in Group K (*p* < 0.05). Detailed measurement data for all serum amino acids are provided in Supplementary Table S4.

### 3.10 Analysis of metabolic substances and microorganisms in the rumen of Tibetan sheep

#### 3.10.1 AA composition and content in rumen fluid

Table 8 demonstrates that the supplementation of Val in the diet influences AA concentrations in the rumen fluid of Tibetan sheep. With increasing Val levels, there is a significant elevation in aspartic acid content (*p* < 0.05). In the control group (K), the rumen of Tibetan sheep showed significantly elevated levels of γ-glutamyl-phenylalanine, creatinine, γ-aminobutyric acid, asparagine, 1-methylhistidine, and hydroxyproline compared to the V1 and V2 groups. Conversely, the concentrations of L-theanine, choline, homoserine, and argininosuccinic acid were significantly lower than those in the V1 and V2 groups. The levels of α-aminobutyric acid and alanine were significantly reduced compared to the V1 group, while aspartylphenylalanine, propionylglycine, and homocitrulline were significantly elevated relative to the V1 group but significantly decreased compared to the V2 group (*p* < 0.05). In the V2 group, the rumen of Tibetan sheep exhibited significantly higher concentrations of N-acetylneuraminic acid, 5-aminolevulinic acid, histidine, N-α-acetyllysine, Leu, and Ile than those in the K and V1 groups. Additionally, levels of leucyl-glycine, tryptophan, proline, and γ-glutamyl-methionine were significantly higher than in the V1 group, whereas taurine levels were significantly lower than in the K group (*p* < 0.005). The K and V2 groups demonstrated significantly elevated concentrations of Val, sarcosine, β-alanine, cis-4-hydroxy-D-proline, glycine, 2,3-diaminopropionic acid, 2,6-diaminopimelic acid, methionine sulfoxide, and N-acetylglutamic acid compared to the V1 group (*p* < 0.05). Detailed measurement data for all rumen fluid amino acids are provided in Supplementary Table S5.

**Table 8:** Effects of valine supplementation on the AA composition and concentration of Tibetan sheep rumen fluid.

Amino acid composition	Amino acid content (ng/mL)			*p*-value
	K	V1	V2	
Homocitrulline	913.78 ± 8.55^b^	649.40 ± 51.29^c^	1402.35 ± 39.20^a^	<0.001
Aspartic acid	8935.61 ± 306.50^c^	10859.70 ± 284.74^b^	12339.77 ± 141.86^a^	0.002
Propionylglycine	108.55 ± 4.15^b^	74.68 ± 5.95^c^	150.76 ± 9.61^a^	0.004
2,6-Diaminopimelic acid	258.10 ± 9.25^a^	197.38 ± 9.51^b^	272.36 ± 1.69^a^	0.005
Aspartylphenylalanine	32.88 ± 1.13^b^	24.49 ± 1.70^c^	46.12 ± 3.76^a^	0.007
Hydroxyproline	270.48 ± 14.87^a^	97.23 ± 19.89^b^	120.18 ± 27.67^b^	0.007
N6-Acetyllysine	265.76 ± 105.08^b^	342.09 ± 3.29^b^	773.56 ± 21.86^a^	0.007
N-Acetylglutamic acid	1882.43 ± 16.68^a^	1149.62 ± 45.48^b^	1965.00 ± 172.30^a^	0.007
Creatinine	95.18 ± 15.79^a^	23.55 ± 0.34^b^	18.54 ± 10.55^b^	0.010
N-Acetylneuraminic acid	11.79 ± 3.71^b^	13.69 ± 0.22^b^	26.39 ± 0.41^a^	0.012
Homoserine	7183.18 ± 954.85^b^	11981.23 ± 289.63^a^	15206.06 ± 1716.16^a^	0.014
L-Theanine	3.53 ± 0.01^b^	4.60 ± 0.38^a^	5.05 ± 0.09^a^	0.015
1-Methylhistidine	20.99 ± 2.64^a^	9.87 ± 0.96^b^	9.74 ± 1.66^b^	0.015
Glycine	29659.77 ± 2910.22^a^	20558.41 ± 1174.97^b^	31620.38 ± 356.77^a^	0.017
Cis-4-Hydroxy-D-proline	406.38 ± 35.75^a^	227.46 ± 30.37^b^	326.26 ± 19.80^a^	0.020
N-α-acetyllysine	387.10 ± 21.00^b^	301.26 ± 37.60^b^	654.50 ± 93.83^a^	0.020
Isoleucine	2498.55 ± 127.66^b^	2526.40 ± 168.80^b^	3131.11 ± 11.96^a^	0.023
γ-Aminobutyric acid	292.36 ± 50.43^a^	137.05 ± 4.92^b^	145.81 ± 18.47^b^	0.026
β-Alanine	19275.02 ± 47.09^a^	14648.34 ± 231.14^b^	18975.55 ± 1625.46^a^	0.028
Asparagine	176.92 ± 10.86^a^	74.95 ± 19.70^b^	101.69 ± 25.17^b^	0.028
Leucine	2917.92 ± 175.78^b^	2802.77 ± 39.28^b^	3542.71 ± 185.92^a^	0.030
Choline	60919.55 ± 5390.47^b^	83282.42 ± 41.06^a^	84996.70 ± 7594.52^a^	0.035
Histidine	171.96 ± 12.62^b^	198.84 ± 15.12^b^	418.60 ± 92.14^a^	0.036
Valine	26261.21 ± 431.03^a^	2735.43 ± 708.44^b^	36031.68 ± 12410.98^a^	0.040
γ-Glutamyl-phenylalanine	418.64 ± 94.77^a^	187.98 ± 6.27^b^	226.14 ± 3.96^b^	0.046
5-Aminolevulinic acid	77.00 ± 22.80^b^	85.32 ± 14.57^b^	281.53 ± 84.40^a^	0.046
Leucyl-glycine	54.20 ± 2.33^ab^	35.83 ± 18.59^b^	83.82 ± 0.57^a^	0.047

The same lowercase letters or no letters indicate no significant difference (*P* > 0.05), and different lowercase letters indicate significant difference (*P* < 0.05).

#### 3.10.2 The pH and SCFAs in rumen fluid

Table 9 details the fermentation characteristics of the rumen in Tibetan sheep subjected to diets with varying levels of Val supplementation. Notable differences in pH values were observed among the three groups, with a significant decrease in rumen fluid pH corresponding to increased Val supplementation levels (*p* < 0.05). The hexanoic acid content in the K group was significantly lower than that in the V1 and V2 groups (*p* < 0.05). Furthermore, the V2 group exhibited a significantly higher butyric acid content compared to the K group (*p* < 0.05), and the levels of isobutyric acid and isovaleric acid in the V2 group were significantly higher than those in the other two groups (*p* < 0.05). However, variations in Val supplementation did not significantly affect the levels of acetic acid, propionic acid, valeric acid, or total SCFAs in the rumen fluid (*p >* 0.05).

**Table 9:** Effects of valine supplementation on the pH and SCFAs of Tibetan sheep rumen fluid.

Index	Groups			*p*-value
	K	V1	V2	
pH	7.31 ± 0.02^a^	7.06 ± 0.03^b^	6.96 ± 0.06^c^	<0.001
Hexanoic acid (ug/mL)	14.69 ± 1.44^b^	25.76 ± 2.28^a^	24.49 ± 3.98^a^	0.050
Butyric acid (ug/mL)	183.79 ± 41.87^b^	267.55 ± 10.31^ab^	296.25 ± 21.08^a^	0.055
Isobutyric acid (ug/mL)	81.20 ± 10.52^b^	95.61 ± 0.85^b^	118.67 ± 4.66^a^	0.025
Isovaleric acid (ug/mL)	65.94 ± 15.35^b^	85.71 ± 0.37^b^	115.95 ± 3.52^a^	0.027
Valeric acid (ug/mL)	54.32 ± 3.61	74.38 ± 2.36	83.85 ± 17.28	0.132
Propionic acid (ug/mL)	281.04 ± 2.44	329.30 ± 33.19	336.06 ± 58.37	0.415
Acetic acid (ug/mL)	650.70 ± 43.57	656.41 ± 25.58	690.45 ± 169.59	0.918
Total SCFAs (ug/mL)	1600.11 ± 15.64	1478.20 ± 41.71	1640.81 ± 158.55	0.339

The same lowercase letters or no letters indicate no significant difference (*P* > 0.05), and different lowercase letters indicate significant difference (*P* < 0.05).

#### 3.10.3 Analysis of rumen microbiota composition

**Figure 6:**
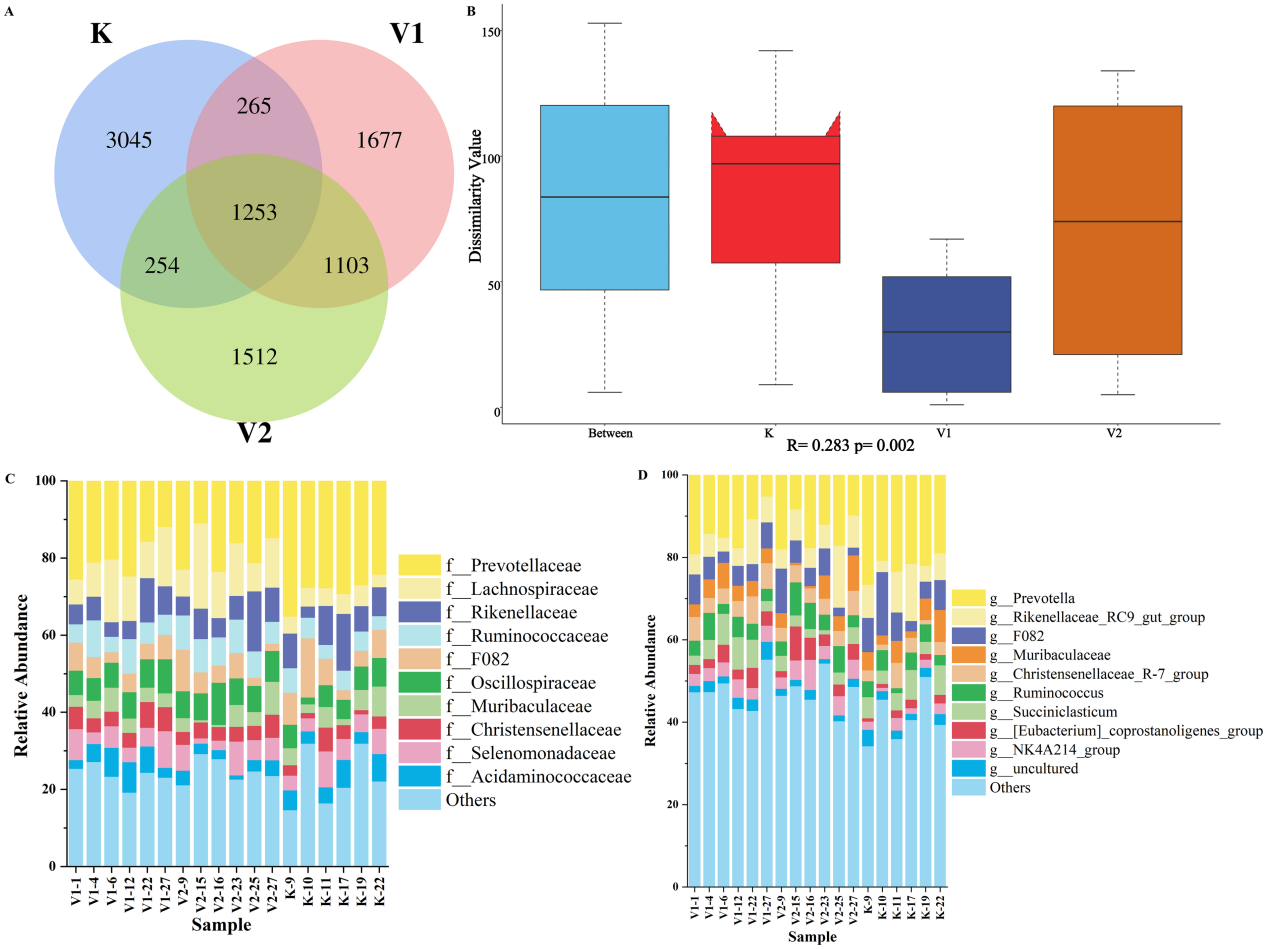
OTU Venn diagrams for rumen microbes across three groups **(A)**. Analysis of variance for total rumen microbial samples **(B)**. Relative abundance of bacterial community proportions at phylum **(C)** and genus **(D)** levels across three sample groups.

As illustrated in Figure 6A, a total of 9,109 operational taxonomic units (OTUs) were identified across the three sample groups, with the K group, V1 group, and V2 group contributing 3,045, 1,677, and 1,512 OTUs, respectively. Notably, significant variations in microbial abundance were detected among these groups, as detailed in Table 10. Specifically, the Shannon index for Group K was significantly lower than those for Groups V1 and V2 (*p* < 0.05), whereas the PD whole tree metric for Group K was significantly elevated compared to Groups V1 and V2 (*p* < 0.05). Furthermore, the Ace and Chao1 indices for Group V1 were significantly higher than those for Group K but significantly lower than those for Group V2 (*p* < 0.05). The Simpson index for Group V2 was significantly greater than that for Group K (*p* < 0.05). PCA plots (Supplementary Figure S2) and Analysis of Similarities (Anosim) plots (Figure 6B) further demonstrated significant differences and distinct separation between Group K and the other two groups, whereas no comparable differentiation was observed between Groups V1 and V2. These results underscore the variations in rumen bacteria communities of Tibetan sheep subjected to three distinct Val-level diets. Additionally, 341 bacterial genera spanning 28 phyla were identified. Figures 6C,D show the top 10 phyla and genera with the highest abundance in each sample.

**Table 10:** Effect of valine supplementation on the alpha diversity of rumen bacteria in Tibetan sheep.

Index	Groups			*p*-value
	K	V1	V2	
Ace	1200.74 ± 74.61^c^	1411.90 ± 96.82^b^	1712.99 ± 91.49^a^	0.001
Chao1	1201.97 ± 79.77^c^	1425.01 ± 93.33^b^	1728.34 ± 105.89^a^	0.001
Simpson	0.98 ± 0.02^b^	0.99 ± 0.01^ab^	0.99 ± 0.00^a^	0.018
Shannon	8.08 ± 0.11^b^	8.90 ± 0.06^a^	8.64 ± 0.34^a^	0.008
PD whole tree	107.07 ± 6.92^a^	75.25 ± 4.00^c^	87.69 ± 4.71^b^	0.001

The same lowercase letters or no letters indicate no significant difference (*P* > 0.05), and different lowercase letters indicate significant difference (*P* < 0.05).

#### 3.10.4 Differential analysis of rumen microorganisms

Table 11 presents the primary differences in rumen bacterial composition among the three groups at both the phylum and genus levels. At the phylum level, Group K exhibited a significantly higher abundance of *p__Spirochaetota* and a significantly lower abundance of *p__Firmicutes* and *p__Actinobacteriota* compared to Groups V1and V2 (*p* < 0.05). Furthermore, as Val concentrations increased, there was a significant decrease in the abundances of *p__Bacteroidota* and *p__Proteobacteria* in the rumen (*p* < 0.05). In comparison to Groups K and V2, Group V1 demonstrated higher levels of *p__Verrucomicrobiota*, *p__Patescibacteria*, and *p__Synergistota*, with *p__Desulfobacterota* and *p__uncultured* also being significantly more abundant in V1 than in V2 (*p* < 0.05). At the genus level, *g__Prevotella* and *g__Rikenellaceae RC9 gut group* were more prevalent in Group K than in Group V1, although no significant difference was observed when compared to Group V2. Groups V1and V2 exhibited higher abundances of the *g__Christensenellaceae R-7 group* compared to Group K. Group V2 showed a higher abundance of *g__Ruminococcus* and *g__[Eubacterium] coprostanoligenes group*, while Group V1 had a higher abundance of *g__Succiniclasticum* and the relative abundance of certain uncultured bacterial taxa (e.g., *g__uncultured*). Additionally, the relative abundance of *g__NK4A214_group* significantly increased with rising Val concentration (*p* < 0.001).

**Table 11:** The main differential rumen bacteria at the phylum and genus levels among the three groups (accounting for the relative abundance in top 10).

Index	Groups			*p*-value
	K	V1	V2	
Phylum level (%)				
*p__Firmicutes*	42.16 ± 1.18^b^	55.10 ± 2.85^a^	55.66 ± 1.56^a^	<0.001
*p__Bacteroidota*	49.83 ± 2.21^a^	39.59 ± 1.56^b^	35.14 ± 0.75^c^	<0.001
*p__Proteobacteria*	4.28 ± 0.15^a^	3.28 ± 0.04^b^	1.74 ± 0.17^c^	<0.001
*p__Verrucomicrobiota*	0.95 ± 0.04^b^	1.34 ± 0.05^a^	1.00 ± 0.08^b^	0.006
*p__Actinobacteriota*	0.47 ± 0.12^b^	1.27 ± 0.26^a^	1.62 ± 0.25^a^	0.002
*p__Patescibacteria*	0.40 ± 0.06^c^	1.53 ± 0.19^a^	1.13 ± 0.12^b^	<0.001
*p__Spirochaetota*	1.01 ± 0.22^a^	0.43 ± 0.01^b^	0.35 ± 0.08^b^	0.006
*p__Desulfobacterota*	0.43 ± 0.05^ab^	0.51 ± 0.03^a^	0.35 ± 0.05^b^	0.019
*p__uncultured*	0.12 ± 0.02^ab^	0.16 ± 0.05^a^	0.07 ± 0.01^b^	0.030
*p__Synergistota*	0.12 ± 0.04^b^	0.26 ± 0.03^a^	0.07 ± 0.02^b^	0.006
Genus level (%)				
*g__Prevotella*	20.50 ± 1.34^a^	17.44 ± 2.04^b^	17.67 ± 0.44^ab^	0.071
*g__Rikenellaceae RC9 gut group*	8.18 ± 1.72^a^	5.57 ± 0.69^b^	7.07 ± 1.13^ab^	0.109
*g__F082*	6.05 ± 1.73	6.34 ± 0.91	7.59 ± 2.81	0.624
*g__Muribaculaceae*	5.01 ± 0.51	4.01 ± 0.47	4.26 ± 1.22	0.353
*g__Christensenellaceae R-7 group*	1.70 ± 0.86^b^	3.74 ± 0.09^a^	3.89 ± 0.23^a^	0.003
*g__Ruminococcus*	4.11 ± 0.24^b^	4.13 ± 0.71^b^	6.91 ± 0.96^a^	0.004
*g__Succiniclasticum*	3.45 ± 0.64^b^	6.34 ± 1.49^a^	3.57 ± 0.55^b^	0.020
*g__[Eubacterium] coprostanoligenes group*	1.75 ± 0.36^b^	2.68 ± 0.73^ab^	3.99 ± 1.29^a^	0.055
*g__NK4A214_group*	2.19 ± 0.37^c^	3.49 ± 0.39^b^	4.66 ± 0.05^a^	<0.001
*g__uncultured*	2.12 ± 0.08^b^	2.75 ± 0.04^a^	1.76 ± 0.22^c^	<0.001

The same lowercase letters or no letters indicate no significant difference (*P* > 0.05), and different lowercase letters indicate significant difference (*P* < 0.05).

#### 3.10.5 Correlation analysis of rumen AAs and SCFAs with differential microorganisms

**Figure 7:**
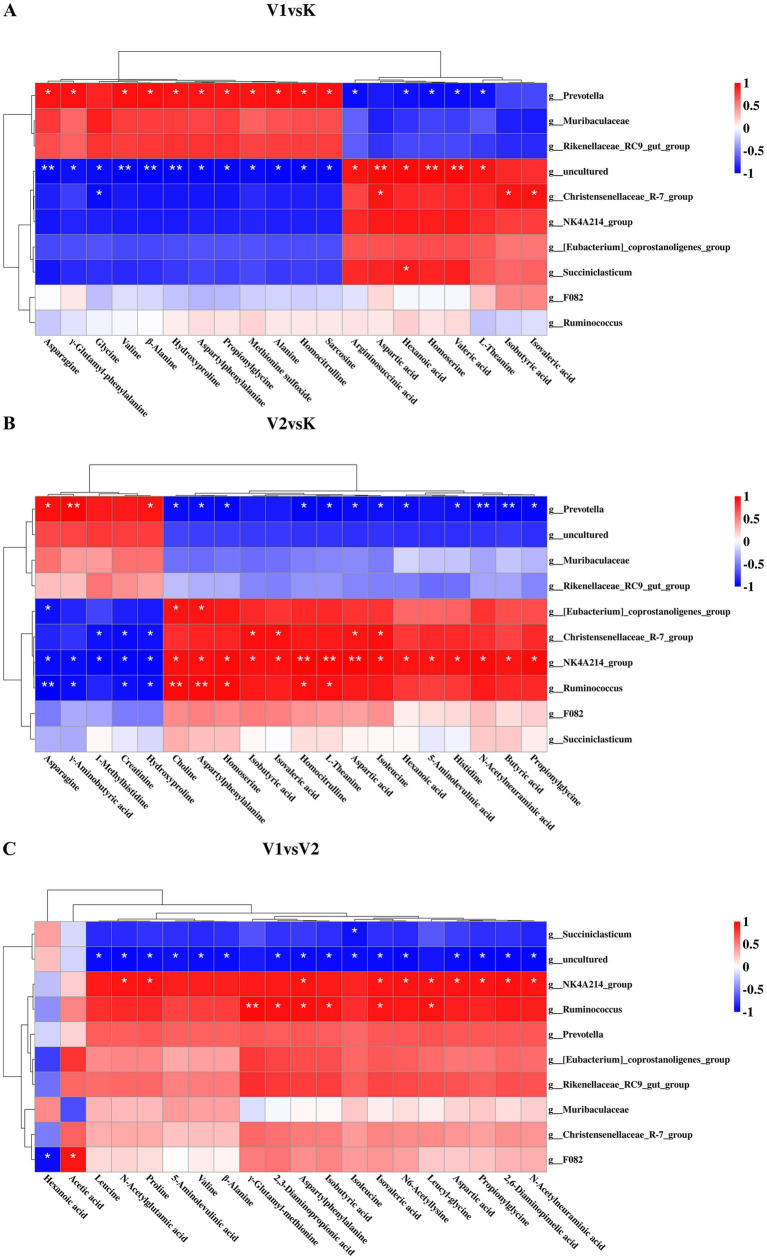
Clustered heatmap of correlations among rumen SCFAs, AAs, and rumen microorganisms. **(A)** Clustering heat map showing the correlation between muscle metabolites and meat quality in V1vsK. **(B)** Clustering heat map showing the correlation between muscle metabolites and meat quality in V2vsK. **(C)** Clustering heat map showing the correlation between muscle metabolites and meat quality in V1vsV2. * indicates *p* < 0.01, ** indicates *p* < 0.001.

To examine the relationship between AAs and SCFAs in the rumen fluid of Tibetan sheep subjected to diets with varying levels of Val and rumen microorganisms at the genus level, a correlation analysis was performed. This analysis assessed the concentrations of AAs and SCFAs in rumen fluid alongside the abundance of rumen microorganisms (Figure 7).

Illustrated in Figure 7. Specifically, Figure 7A presents a correlation clustering heatmap that delineates the associations between rumen AAs and SCFAs and rumen microorganisms within the V1 and K groups, identifying 21 positive and 18 negative correlations (*p* < 0.01). The genus *Prevotella* exhibited negative correlations with L-theanine, argininosuccinic acid, homocitrulline, aspartic acid, hexanoic acid, and valeric acid, while showing positive correlations with other differentially expressed rumen AAs. Conversely, the genera *Christensenellaceae_R-7_group* and certain uncultured bacterial taxa (e.g., *g__uncultured*) demonstrated positive correlations with homocitrulline, L-theanine, aspartic acid, hexanoic acid, valeric acid, isobutyric acid, and isovaleric acid, and negative correlations with other differentially expressed AAs (*p <* 0.01). Figure 7B illustrates a correlation clustering heatmap that delineates the associations between rumen AAs and SCFAs with rumen microbiota in the V2 and K groups. This analysis identifies 29 positive correlations and 25 negative correlations, all statistically significant (*p <* 0.01). Notably, the genus *Prevotella* demonstrated positive correlations with γ-aminobutytic acid, asparagine and 1-methylhistidine, while showing negative correlations with butyric acid, propionylglycine, N-acetylneuraminic acid, aspartic acid and differentially expressed rumen AAs. Conversely, the genera *Christensenellaceae_R-7_group*, *Ruminococcus*, *[Eubacterium]_coprostanoligenes_group*, and *NK4A214_group* exhibited correlations opposite to those of *Prevotella* (*p* < 0.01). Figure 7C provides a correlation clustering heatmap depicting the relationships between rumen AAs and SCFAs with rumen microbiota in Tibetan sheep from the V1 and V2 groups, identifying 17 positive correlations and 18 negative correlations (*p <* 0.01). The genus *Succiniclasticum*, along with certain uncultured bacterial taxa (e.g., *g__uncultured*), displayed negative correlations with the majority of differentially expressed rumen SCFAs and AAs; the genus *Ruminococcus* and *NK4A214_group* showed positive correlations with most differentially expressed rumen SCFAs and AAs; *F082* showed a negative correlation with hexanoic acid and positive correlations with acetic acid (*p <* 0.05).

### 3.11 Correlation analysis of meat quality with rumen AAs, SCFAs, and microorganisms

**Figure 8:**
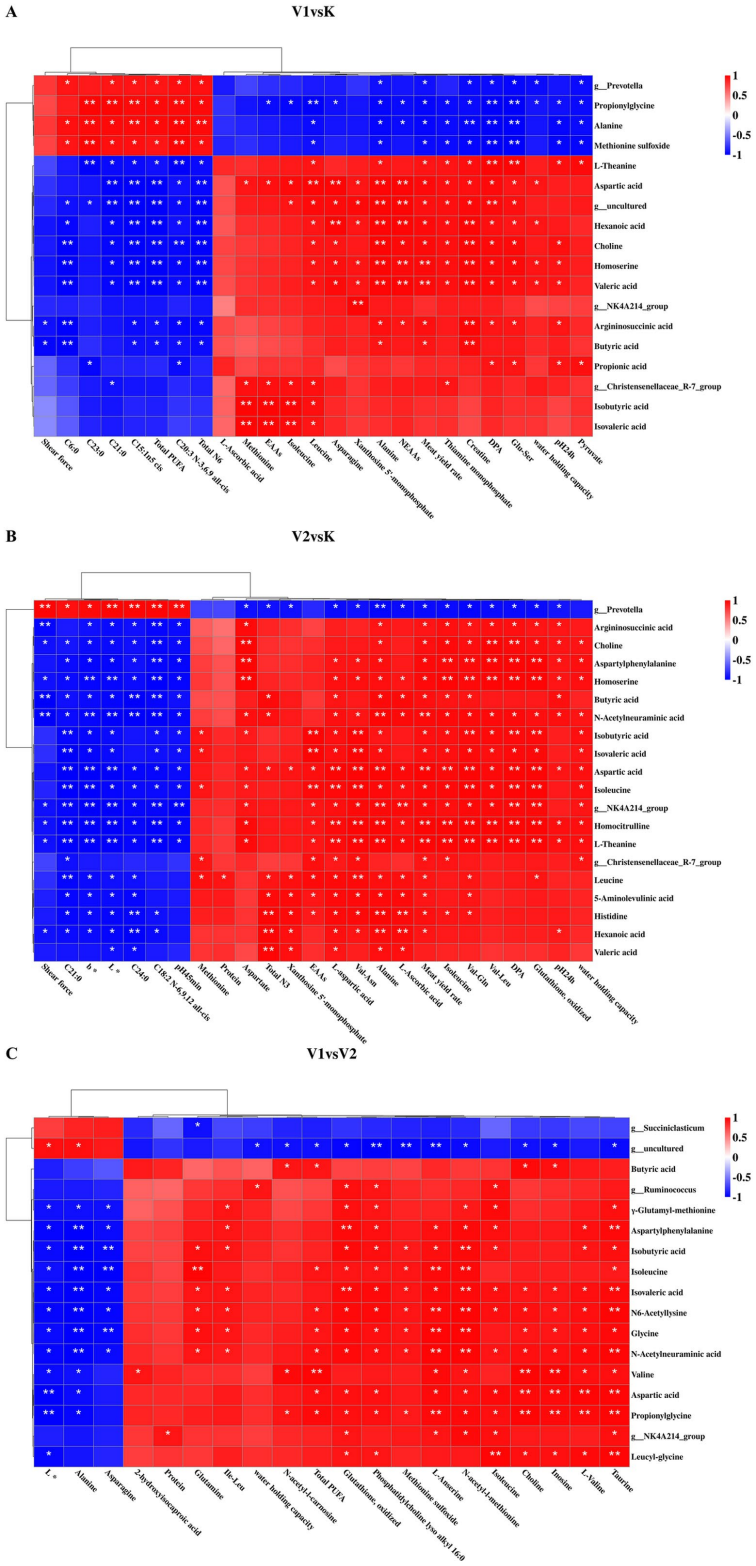
Clustered heatmap of correlations among meat quality, rumen AAs, SCFAs, and rumen microorganisms. **(A)** Clustering heat map showing the correlation between muscle metabolites and meat quality in V1vsK. **(B)** Clustering heat map showing the correlation between muscle metabolites and meat quality in V2vsK. **(C)** Clustering heat map showing the correlation between muscle metabolites and meat quality in V1vsV2. * indicates *p* < 0.01, ** indicates *p* < 0.001.

To examine the interrelationships among meat quality, muscle metabolites, rumen microbiota, and SCFAs in Tibetan sheep subjected to diets with varying levels of Val supplementation, correlation analyses were performed on phenotypic meat data, untargeted metabolomics findings, rumen microbial genus levels, and rumen AAs and SCFAs content. As illustrated in Figure 8, these variables demonstrated a robust correlation.

Specifically, Figure 8A presents a correlation clustering heatmap that delineates the associations among meat quality, muscle metabolites, rumen microbiota, AAs and SCFAs in the V1 and K groups of Tibetan sheep. Notably, negative correlations were identified between shear force and the genus *Succiniclasticum*, *NK4A214_group*, choline, homoserine, argininosuccinic acid, hexanoic acid, valeric acid, and butyric acid. Furthermore, L-ascorbic acid exhibited negative correlations with *g__Prevotella*, sarcosine, alanine, homoserine, and methionine sulfoxide, while showing positive correlations with L-theanine and propionic acid. Pyruvate was positively correlated with certain uncultured bacterial taxa (e.g., *g__uncultured*), L-theanine, choline, homoserine, argininosuccinic acid, aspartic acid, hexanoic acid, isobutyric acid, valeric acid, and propionic acid (*p* < 0.05). Figure 8B displays a correlation clustering heatmap that elucidates the relationships among Tibetan sheep meat quality, muscle metabolites, rumen microbiota, AAs and SCFAs in the V2 and K groups. Notable correlations were identified between shear force and various compounds, including *g__NK4A214_group*, aspartylphenylalanine, L-theanine, N-acetylneuraminic acid, choline, 5-aminolevulinic acid, homoserine, histidine, argininosuccinic acid, Leu, Ile, homocitrulline, aspartic acid, hexanoic acid, butyric acid, isobutyric acid, and valeric acid (*p* < 0.05). Figure 8C similarly presents a correlation clustering heatmap, illustrating the relationships among Tibetan sheep meat quality, muscle metabolites, rumen microbiota, AAs and SCFAs in the V1 and V2 groups. Significant correlations were observed between water content and several compounds, including aspartylphenylalanine, N-acetylneuraminic acid, leucylglycine, tryptophan, glycine, propionylglycine, 2,3-diaminopropionic acid, 2,6-diaminopimelic acid, N6-acetyllysine, isoleucine, aspartic acid, γ-Glutamyl-methionine, isobutyric acid, isovaleric acid, and *g__Ruminococcus*. Chromaticity, L*, alanine, asparagine, and NEAAs showed negative correlations with most rumen microorganisms, AAs, and SCFAs, while exhibiting positive correlations with *g__Succiniclasticum* and certain uncultured bacterial taxa (e.g., *g__uncultured*) (*p* < 0.05).

## 4 Discussion

Previous research by our team (Jia et al., 2025; Ma et al., 2025) revealed that Tibetan sheep fed on saline-alkali pasture grass exhibited superior meat quality. Subsequent analysis of grasses from saline-alkali and non-saline-alkali regions showed significantly higher valine content in saline-alkali pasture grass. Therefore, this study supplemented the basal diet with valine to investigate its effects on Tibetan sheep meat quality and rumen microbiota. Based on Roth-Maier et al. (2004), valine supplementation levels were set at 0, 0.10, and 0.15%. In well-balanced diets, when valine becomes a limiting factor, its supplementation synergizes with key amino acids like lysine and methionine to enhance overall protein deposition efficiency (Corzo et al., 2008). L-valine is a naturally occurring physiological component in both animal and plant organisms. L-valine added to feed will be incorporated into animal-derived tissue proteins and/or animal-derived products, with excess amounts metabolized and excreted. Therefore, the use of L-valine in animal nutrition does not affect the protein composition of animal tissues and products. Consequently, the Panel concludes that the use of this additive in animal nutrition is safe for consumers (EFSA Panel on Additives and Products or Substances used in Animal Feed (FEEDAP) et al., 2025).

This study demonstrated that dietary supplementation with Val in Tibetan sheep resulted in increased meat yield, rib thickness, abdominal wall thickness, and backfat thickness. Concurrently, alterations in meat quality parameters were observed, primarily reflected in changes in pH (at 45 min and 24 h), L*, a*, b* values, and tenderness, etc. *p_Firmicutes, p_Actinobacteriota,* and *g_Christensenellaceae R-7 group* were more prevalent in the V1 and V2 groups compared to the K group. The markedly reduced abundance of *Bacteroidetes* and *Proteobacteria* in the V1 and V2 groups, as compared to the K group. The elevated concentrations of isobutyric and isovaleric acids observed in the V2 group. We propose the hypothesis that different levels of valine supplementation may alter the abundance of *p_Firmicutes, p_Actinobacteriota*, and *g_Christensenellaceae R-7 group* in Tibetan sheep rumen microbiota, as well as the levels of isobutyric acid, isovaleric acid, leucine, aspartic acid, and others in rumen fluid. This could influence serum amino acid content, thereby regulating the spectrum of absorbed amino acids in muscle tissue. Consequently, it may modulate muscle energy metabolism and protein deposition, ultimately enhancing Tibetan sheep meat quality. Interactions among these factors are possible.

Ruminants depend on microbial fermentation within the rumen to digest plant fiber and convert nutrients that are otherwise indigestible into absorbable animal proteins (Chen et al., 2025). Rumen microbes, often referred to as the “second genome,” play a pivotal role in influencing host physiological functions and meat quality through their metabolic products (Wang et al., 2021). They are essential for ruminant digestion, with variations in their abundance and community composition reflecting changes in feed and rumen function, thereby impacting meat quality (Ma et al., 2023; Guo et al., 2024). Certain rumen microbes have been associated with the accumulation of AAs deposition of AAs and lipid metabolites in muscle tissue (Wang et al., 2021). Zhang C. et al. (2025) demonstrated that *Firmicutes*, *Bacteroidetes*, and *Proteobacteria* are the predominant rumen microbiota in ruminants, a finding that aligns with the results of the present study. *Bacteroidetes* are involved in the degradation of non-fiber substances, facilitating the digestion and absorption of proteins and non-cellulose polysaccharides. These non-fiber substances are converted into propionic acid, which is utilized for bacterial protein synthesis and enhances AA absorption (Zheng et al., 2024). In this study, *Firmicutes* and *Actinobacteriota* were more prevalent in the V1 and V2 groups compared to the K group, which may account for the upregulation of metabolic pathways observed in the V1 and V2 groups relative to the K group. The markedly reduced abundance of *Bacteroidetes* and *Proteobacteria* in the V1 and V2 groups, as compared to the K group, indicates that valine supplementation may improve protein digestion and absorption while mitigating inflammatory responses in Tibetan sheep. Nonetheless, the precise mechanisms driving these effects warrant further exploration. *Prevotella* and *Rikenellaceae_RC9_gut_group* were predominant in the rumen of Tibetan sheep (Liu et al., 2020), aligning with the findings of the present study. *Succiniclasticum* is known to convert succinate into propionate (Lv et al., 2019). The significantly increased abundance of *Succiniclasticum* in the V1 group resulted in elevated propionic acid levels in the rumen of Tibetan sheep. Higher concentrations of acetic acid provided additional substrates for amino acid synthesis in the rumen, while increased propionic acid levels supplied substantial energy to the Tibetan sheep (Nathani et al., 2015), ultimately enhancing the functional productivity of rumen microorganisms in the V1 and V2 groups. The *Christensenellaceae R-7 group* is capable of fermenting dietary fiber to produce SCFAs, particularly acetate and butyrate (Zimmer et al., 2021). Its abundance in the V1 and V2 groups was significantly higher than in the K group, which likely contributed to the increased levels of rumen acetate and butyrate observed in these groups compared to the K group. As Val levels increase, the abundance of the *g__NK4A214_group* also rises significantly, indicating that Val may contribute to a healthier rumen ecosystem in Tibetan sheep. This ecosystem is capable of efficiently producing microbial crude protein (MCP) and SCFAs, ultimately providing the foundation for muscle growth and development. Correlation analysis (Figure 7) demonstrated a positive correlation between the *NK4A214_group* and the concentrations of propionic acid, isobutyric acid, and isovaleric acid, aligning with findings from previous studies (Liu et al., 2022; Pang et al., 2022). The relative abundance of this genus increased with higher Val levels, which may also account for the elevated concentrations of isobutyric and isovaleric acids observed in the V2 group. Although the rumen valeric acid content, a metabolite of branched-chain amino acids (such as valine), increased with higher valine supplementation, this increase was not statistically significant. This suggests that valine levels have a minimal impact on rumen SCFA concentrations.

Amino acids (AAs) are essential components that enhance the flavor and nutritional value of lamb (Zhou et al., 2024), playing a crucial role in regulating growth, immune function, and metabolic pathways. AAs in the rumen primarily originate from the degradation of dietary protein and the synthesis by rumen microorganisms (Guo et al., 2022). Notably, a substantial portion of the bacterial cell protein synthesized by rumen microorganisms is digested and absorbed in the small intestine, serving as a vital AA source for ruminants (Zhou et al., 2025). Serum amino acid (AA) levels are generally influenced by AA content in feed and can partially reflect dietary AA composition as well as metabolic processes within the organism. They are widely considered a key indicator for assessing AA metabolism (Hu et al., 2022). Serum AA concentrations sever as vital indicators for examining AA balance patterns. AAs absorbed from the intestine enter the bloodstream and are utilized in conjunction with endogenous AAs for processes such as protein synthesis, degradation, or conversion into other compounds (Correa et al., 2024). Branched-chain amino acid (BCAA) catabolism plays a pivotal role as a nitrogen source for AA synthesis. This metabolic process is facilitated by BCAA transaminases, which catalyze the transamination of AAs such as glutamate and aspartate, thereby enhancing protein synthesis (Li et al., 2025). BCAAs facilitate muscle growth by enhancing glucose uptake and utilization through the upregulation of glucose transporters (Wang et al., 2025). This aligns with the findings of the present study, where the V2 group demonstrated increased upregulation of the glucuronide biosynthesis pathway and exhibited reduced shear force in comparison to the V1 group. Consequently, we propose the hypothesis that elevated levels of valine may impact aspartate concentrations in the rumen of Tibetan sheep. Serum aspartic acid levels increased significantly with higher valine concentrations, potentially leading to L-aspartic acid enrichment in muscle tissue. Serine can be directly converted to pyruvate via the action of serine dehydratase (Zhang B. et al., 2025), which likely accounts for the upregulation of pyruvate metabolites in muscle tissue. Furthermore, betaine plays a role in methyl metabolism by providing methyl groups for essential physiological reaction. Sufficient availability of methyl groups is essential for the normal synthesis of phospholipids, neurotransmitters, and other compounds, thereby regulating systemic energy metabolism and facilitating the deposition of intramuscular fat (Yang et al., 2022). The content of intramuscular fat is a critical determinant of meat tenderness, flavor, and juiciness (Jin et al., 2024). Following valine supplementation, there was a significant increase in serum betaine levels, which may account for the upregulation of L-methionine metabolites and intramuscular fat in muscle tissue, consequently enhancing methionine content in the *longissimus dorsi* muscle. Furthermore, it was observed that varying levels of Val supplementation in the feed increased the deposition of EAAs in the dorsal *longissimus muscle* of Tibetan sheep. Consequently, the muscle of Tibetan sheep fed with valine-supplemented feed exhibits a higher nutritional value of AAs, thereby offering greater benefits for human health.

Protein is crucial for normal biological development and serves as the primary source of EAAs (Borba et al., 2022). The studies have indicated that activation of the AKT/mammalian target of rapamycin (mTOR) pathway and enhancement of S6K1 phosphorylation activity can regulate protein deposition in muscle (Liang et al., 2022). In this study, the protein content in the V1 and V2 groups was significantly higher than in the K group, and an increase in protein content in Tibetan sheep muscle was observed with elevated Val supplementation. In the correlation analyses presented in Figures 5, 8, the protein content in the *dorsal longissimus* muscle of Tibetan sheep exhibited a positive correlation with the metabolites inosine monophosphate (IMP), L-ascorbic acid, L-aspartic acid, and xanthosine 5′-monophosphate (XMP). Conversely, it demonstrated a negative correlation with the SCFAs in the rumen. Previous research suggests that SCFAs, particularly butyric acid and propionic acid, may act as signaling molecules that directly or indirectly influence cellular proliferation and anabolic metabolism by modulating key signaling pathways such as mTOR (Frampton et al., 2020). IMP and XMP, recognized as umami nucleotides, are not only essential components of meat flavor but also serve as intermediate metabolites in amino acid biosynthesis and metabolism (Deng et al., 2022). IMP functions as a substrate for the synthesis of adenosine triphosphate (ATP) and guanosine triphosphate (GTP) (Hernandez et al., 2024), with ATP providing direct energy for protein synthesis (AA activation and peptide chain elongation). Therefore, we hypothesize that the upregulation of IMP and XMP in muscle provides the foundation for protein deposition. L-ascorbic acid is a powerful antioxidant that antioxidant that neutralizes free radicals, mitigates inflammation, and safeguards cells against oxidative damage (Ma et al., 2024). Concurrently, it functions as an essential cofactor for prolyl hydroxylase and lysyl hydroxylase, enzymes critical for collagen biosynthesis (Nassar et al., 2025). Collagen, as the predominant constituent of muscle connective tissue, plays a pivotal role in determining total protein content and meat tenderness through its synthesis (Shi et al., 2024). L-aspartic acid is a vital amino acid in the urea cycle and purine nucleotide synthesis, supplying abundant substrates for protein synthesis. Histidine, leucine, isoleucine, and aspartic acid are either essential AAs and hold significant functional importance in animals. Leucine, in particular, has been shown to regulate muscle protein synthesis by activating the mTOR pathway, thereby enhancing protein synthesis at the translational level (Goo and Kim, 2025). Their elevated concentrations in rumen fluid suggest increased availability of high-quality AAs for absorption in the small intestine and subsequent muscle protein synthesis.

Tenderness is a vital indicator for evaluating lamb quality in edible products, closely associated with consumer purchasing decisions and satisfaction (Vidal et al., 2025). Shear force is the most prevalent method for quantifying meat tenderness, representing the force exerted by teeth during mastication and showing an inverse relationship with meat tenderness (Xiong et al., 2022). Meat tenderness is influenced by multiple factors, including collagen and fat content in muscle tissue, sarcomere length, and the rate of pH decline (Veselá et al., 2024). A rapid decrease in pH induces muscle contraction, thereby diminishing tenderness (Zhang et al., 2023). Postmortem hypoxia in muscle cells impedes significant ATP production through the citric acid cycle and oxidative phosphorylation, gradually shifting ATP generation toward glycolysis. This metabolic shift results in lactic acid accumulation and a consequent decrease in pH (Warner et al., 2022). Group K exhibited a pH value of 7.07 at 45 min, which was higher than that of the other two groups. After 24 h, the pH of Group K decreased to 5.77, significantly lower than that of the other groups, indicating a rapid pH decline in the muscle of Group K. This may explain why Group K exhibits significantly greater tenderness than V1 and V2. Hypoxanthine functions as a crucial intermediate in both the synthesis and degradation of purine nucleotides, specifically adenosine monophosphate (AMP) and guanosine monophosphate (GMP), effectively linking the essential pathways of “purine synthesis” and “purine recycling” (Fang et al., 2025). Similarly, 5-ALA acts as an intermediate in the heme synthesis pathway, facilitating the conversion from succinyl-CoA to heme (Takada et al., 2022). Post-mortem muscle tissue is subject to oxidative stress, with lipid and protein oxidation being primary contributors to the deterioration of meat quality, manifesting as reduced tenderness, moisture loss, and off-flavor (Mussa et al., 2025). Oxidative processes may lead to protein cross-linking, which results in a tougher muscle structure. Consequently, we propose that the upregulation of 5-ALA in muscle tissue may enhance heme synthesis, thereby augmenting the antioxidant capacity of muscle cells. This, in turn, mitigates oxidative damage to proteins post-slaughter and helps preserve meat tenderness. Amono acid (AA) metabolism can produce pyruvate (Wang et al., 2024). Following Val supplementation in feed, the conversion of phosphoenolpyruvate to pyruvate via upregulation of amino acid metabolic pathways and the phosphotransferase system (PTS). Subsequently, pyruvate inhibits gluconeogenesis and cyclic adenosine monophosphate (cAMP) production, further suppressing glycolysis (Zhang et al., 2023; Ma et al., 2023). This mechanism delays the decline in pH, reduces myofibrillar contraction, and enhances muscle tenderness. Therefore, we hypothesize that Val supplementation in the V1 and V2 groups may reduce cAMP levels and inhibits glycolysis by upregulating PTS and amino acid metabolism pathways. This modulation of pH decreased in the V1 and V2 groups, thereby improving the tenderness of the *longissimus dorsi* muscle. Subsequent analyses will focus on transcriptomics and proteomics to identify differentially expressed genes and proteins. Key genes and differentially expressed proteins will be validated using PCR and Western blot (WB) techniques.

**Figure 9:**
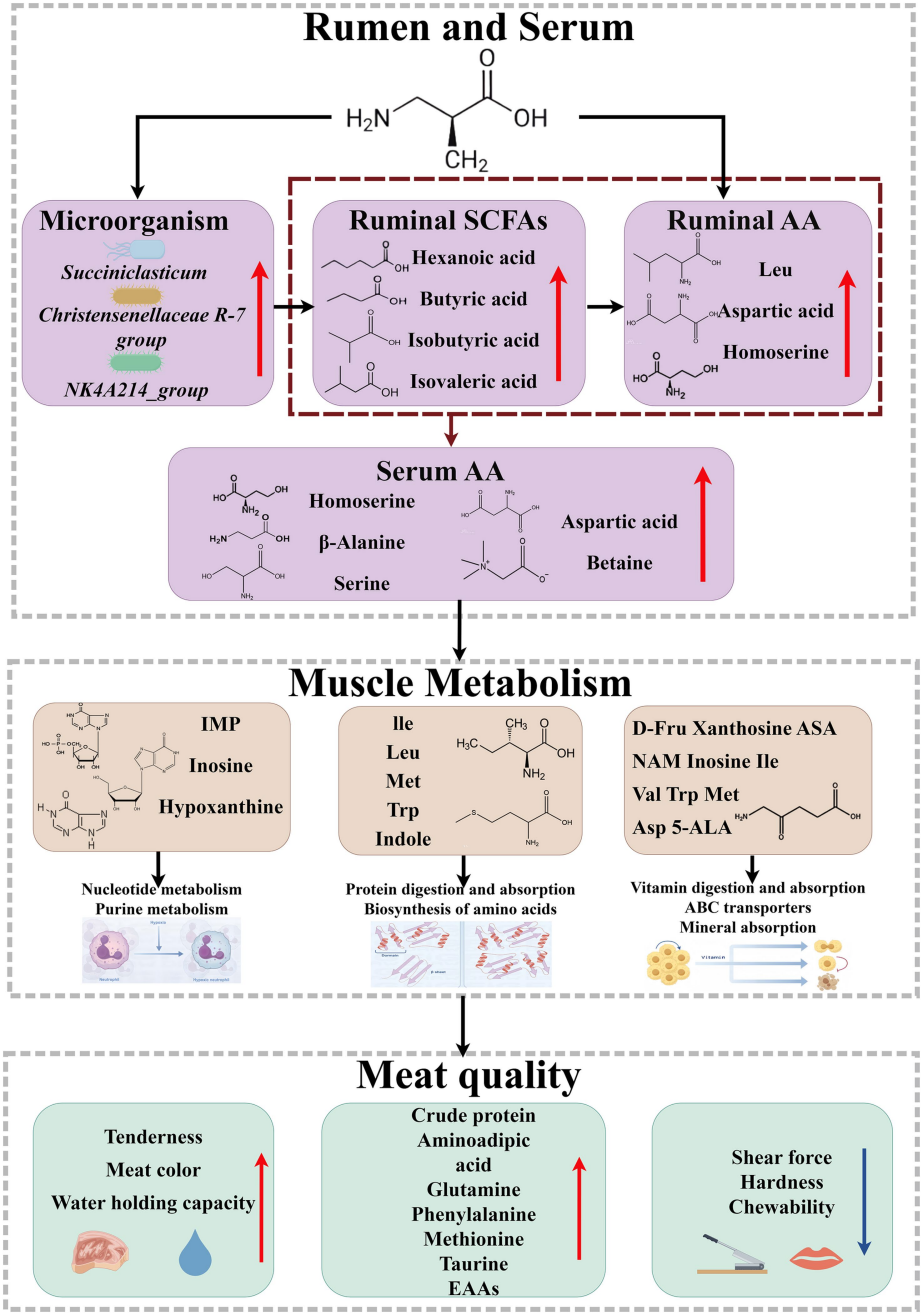
Hypothesized pathways and potential mechanisms associated with Val on muscle metabolomics, rumen microbiology, and meat quality changes (by Figdraw).

In conclusion, our findings suggest that Val initially may modulate the abundance of microbial genera, including *Succiniclasticum*, *Christensenellaceae R-7 group*, and *NK4A214_group*, within the rumen of Tibetan sheep. This modulation subsequently impacts rumen fluid SCFAs, such as propionic acid, isobutyric acid, and isovaleric acid, as well as rumen AAs including leucine, aspartic acid, homoserine, and creatinine. These changes further influence serum concentrations of creatinine, homoserine, β-hydroxybutyrate, myosin, hydroxyproline, and threonine. Collectively, these factors affect muscle metabolites, including pyruvate, L-aspartic acid, L-ascorbic acid, isoleucine, indole, DL-tryptophan, L-methionine, and L-valine, thereby influencing metabolic pathways related to amino acid biosynthesis and protein digestion and absorption. As a result, these processes may impact the growth performance of Tibetan sheep and affect meat quality attributes such as tenderness, pH value, and color parameters (a* and b* values) (Figure 9). Nevertheless, further research is necessary to elucidate the underlying mechanisms in greater detail.

## 5 Conclusion

Research first reveals that varying levels of valine supplementation may synergistically regulate rumen microbiota (such as *Christensenellaceae R-7* and *NK4A214 groups*) by altering rumen fermentation patterns, while simultaneously affecting rumen amino acid and short-chain fatty acid levels, ultimately influencing serum and muscle amino acid metabolic networks. These effects may upregulate phosphosphatidylserine (PTS) and amino acid biosynthesis pathways in muscle tissue, thereby slowing the post-slaughter pH decline rate and oxidative stress levels in the *longissimus dorsi* muscle. This enhances meat yield, tenderness, and color in Tibetan sheep while promoting protein deposition within muscle tissue. In summary, valine plays a crucial role in maintaining meat color, improving tenderness, and enhancing protein deposition in Tibetan sheep muscle. Provide a theoretical basis for precision nutritional regulation in Tibetan sheep.

## Data Availability

The data presented in this study are publicly available. The data can be found here: https://www.ncbi.nlm.nih.gov, accession PRJNA1418505.

## Footnotes

1
http://www.genome.jp/kegg
