# Supplementary material for: Effects of varying levels of valine supplementation on meat metabolism and rumen microorganisms in Tibetan sheep
Source: Front Microbiol. 2026 Mar 27;17:1806032. doi: 10.3389/fmicb.2026.1806032 (PMC13066278; doi:10.3389/fmicb.2026.1806032)
Supplement: Supplementary file 1 [file supplementary_file_1.docx]

# Supplementary Figures and Tables

## Supplementary Figures


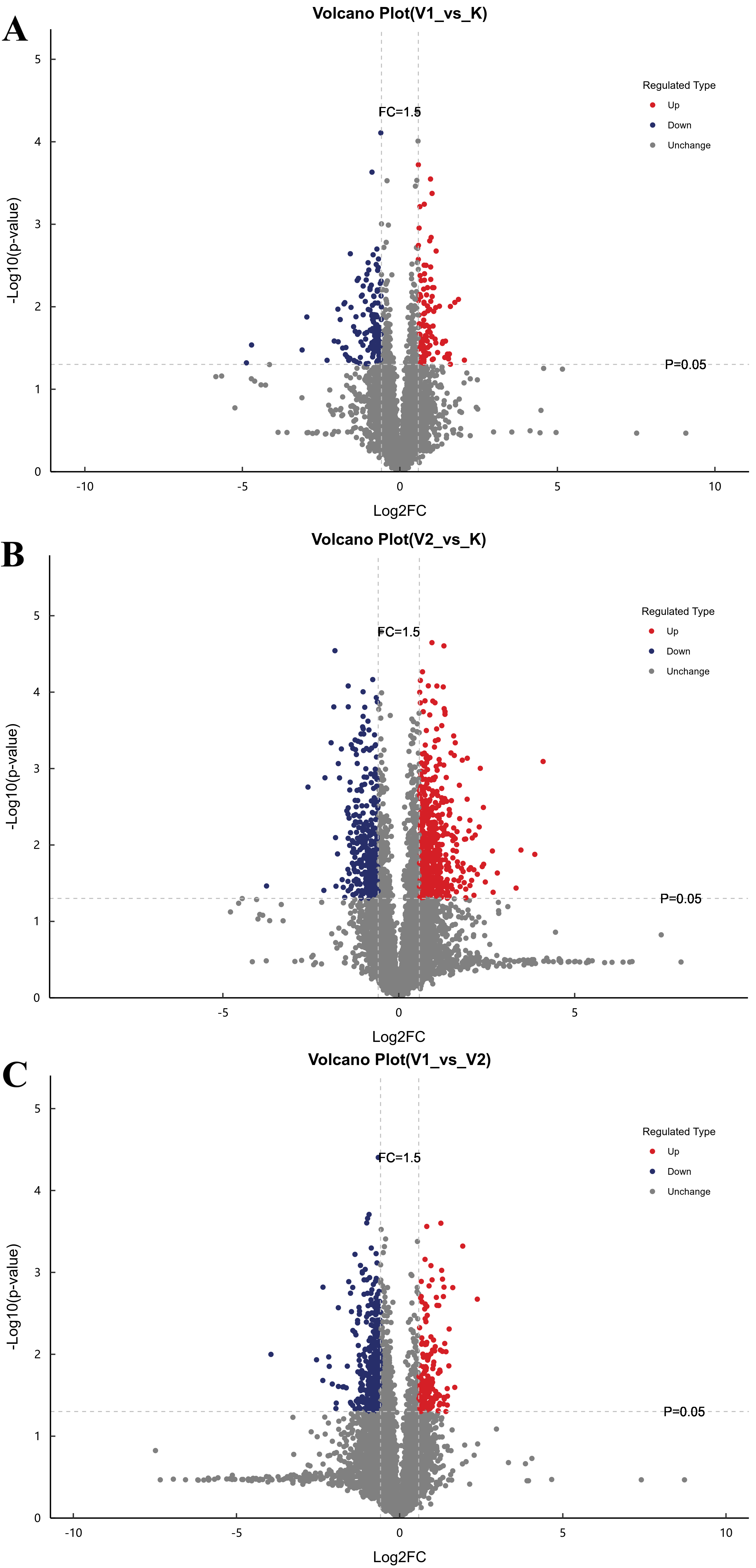


Supplementary Figure 1 Volcano plot of muscle metabolites in Tibetan sheep fed different levels of Val.

Note: A-C: Volcano plots of V1 vs K, V2 vs K, and V1 vs V2, respectively, in negative ion mode.


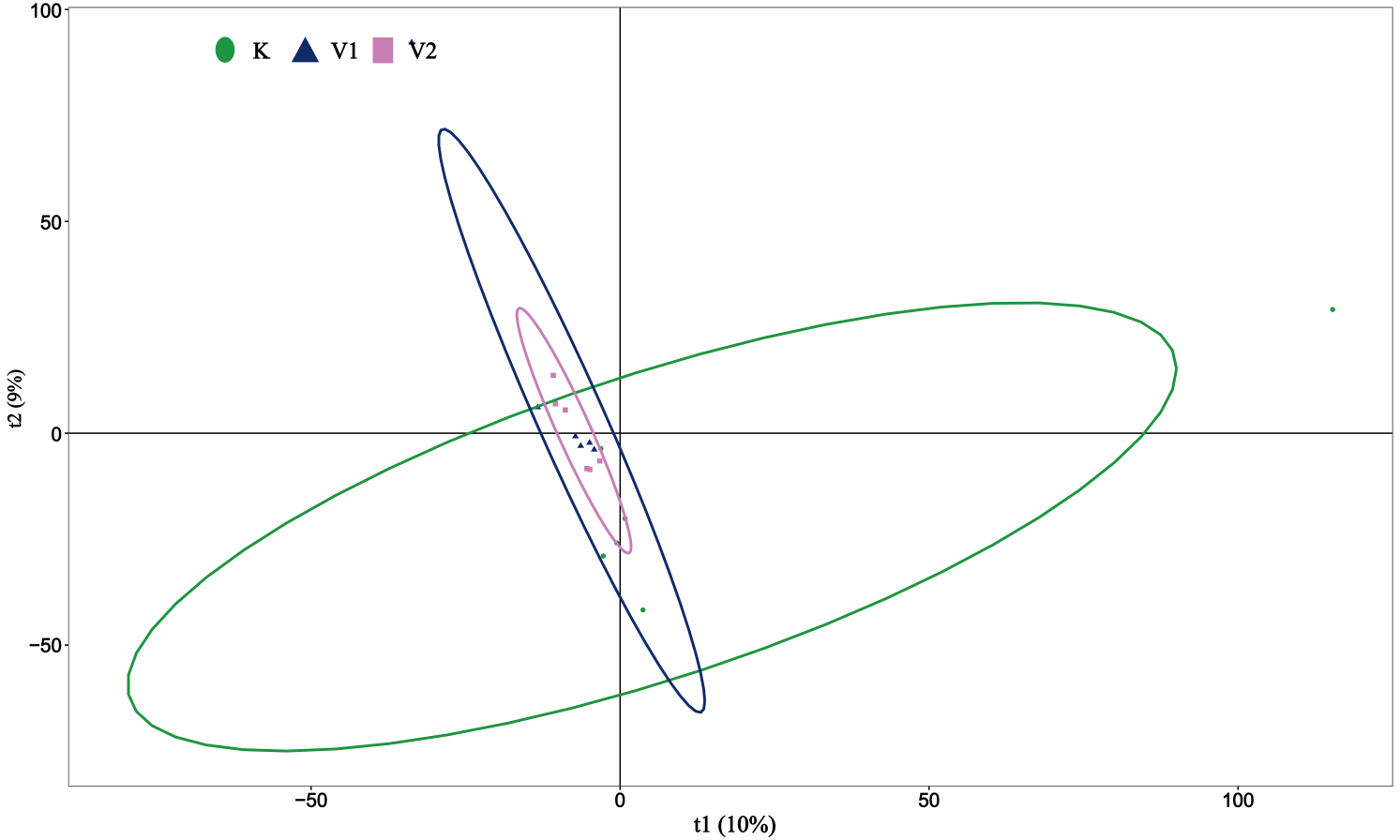


Supplementary Figure 2 Principal Component Analysis (PCA) Plot of Total Rumen Microbial Samples.

## Supplementary Tables

Supplementary Table 1 Sensory scoring criteria for meat samples.

| Evaluation project | Grading Standards | Score |
| --- | --- | --- |
| Organizational status | Regular appearance, small gaps, fibrous structure. | 15-20 |
|  | Appearance is relatively regular with small voids; fibrous structure is present but not prominent. | 10-14 |
|  | Irregular appearance, with gaps of varying sizes, lacking fibrous structure. | 0-9 |
| Elasticity | The meat is tough and resilient, with good elasticity; when pressed with a finger, it indents but then springs back. | 15-20 |
|  | The texture is tough and resilient, with a certain degree of elasticity. When pressed, it takes a bit longer to spring back. | 10-14 |
|  | Poor elasticity of the flesh; indentations remain after pressure is applied. | 0-9 |
| Flavor | Possesses the distinctive flavor of lamb, with no off-flavors whatsoever. | 15-20 |
|  | Lacks distinctive flavor, with slight off-flavors such as ammonia, sourness, or rancidity. | 10-14 |
|  | The meat has a strong, unpleasant odor and is unacceptable. | 0-9 |
| Palatability | Delicately textured with a satisfying chew, it doesn't feel dry when chewed. | 15-20 |
|  | The texture is somewhat coarse with a slight chewiness, feeling either soft or firm when chewed. | 10-14 |
|  | The texture is coarse and crumbly, feeling dry when chewed, or alternatively very soft or very hard. | 0-9 |
| Overall evaluation | Overall, it feels great. | 15-20 |
|  | Overall, it feels good. | 10-14 |
|  | Overall, it feels subpar. | 0-9 |

Supplementary Table 2 Effect of valine supplementation on the AA content and composition of Tibetan sheep.

| Amino acid composition | AA content (umol/kg) | | | *p-value* |
| --- | --- | --- | --- | --- |
|  | K | V1 | V2 |  |
| Aminoadipic acid | 25.64±0.42^c^ | 38.21±0.44^b^ | 69.32±1.46^a^ | <0.001 |
| Ornithine | 14.40±0.17^a^ | 9.79±0.39^b^ | 9.57±0.10^b^ | <0.001 |
| Alanine | 3679.35±11.36^c^ | 4102.96±40.06^a^ | 3844.92±24.62^b^ | 0.001 |
| Glutamine | 221.99±3.94^c^ | 392.71±45.37^b^ | 492.36±19.78^a^ | 0.006 |
| Phenylalanine | 112.59±8.04^b^ | 160.40±4.39^a^ | 153.22±7.16^a^ | 0.011 |
| Choline | 17710.08±150.60^a^ | 10872.15±687.23^b^ | 15184.45±1565.82^a^ | 0.014 |
| Taurine | 3046.56±132.43^b^ | 2782.00±225.71^b^ | 3807.60±55.75^a^ | 0.014 |
| Leucine | 391.04±25.07^b^ | 488.08±0.35^a^ | 438.10±18.46^ab^ | 0.028 |
| Cysteine | 0.74±0.05^a^ | 0.27±0.15^b^ | 0.16±0.14^b^ | 0.035 |
| Methionine | 36.89±12.82^b^ | 57.34±2.20^a^ | 57.24±4.78^a^ | 0.035 |
| Asparagine | 202.66±4.20^b^ | 270.43±22.04^a^ | 194.89±18.32^b^ | 0.036 |
| Glutamate | 346.37±14.47^a^ | 244.78±44.31^ab^ | 184.93±36.36^b^ | 0.039 |
| Creatine | 4151.26±58.05^b^ | 4413.10±2.42^a^ | 4305.21±80.20^ab^ | 0.044 |
| Isoleucine | 100.53±18.11^b^ | 142.03±0.31^a^ | 126.39±1.00^ab^ | 0.063 |
| Threonine | 224.03±6.80^b^ | 275.40±17.89^a^ | 226.87±19.00^ab^ | 0.076 |
| Aspartate | 134.10±34.21^b^ | 243.01±48.45^ab^ | 337.49±77.00^a^ | 0.080 |
| Hydroxyproline | 67.09±4.76 | 71.91±2.25 | 56.65±7.63 | 0.133 |
| Proline | 78.96±4.38 | 84.28±3.80 | 73.01±3.38 | 0.134 |
| Histidine | 924.90±52.37 | 963.33±6.22 | 1030.22±38.12 | 0.141 |
| Glycine | 1094.51±35.09 | 1355.63±125.86 | 1038.99±172.49 | 0.157 |
| Tyrosine | 79.28±3.72 | 130.41±19.40 | 82.51±32.82 | 0.172 |
| Lysine | 174.68±1.36 | 170.01±38.44 | 244.03±48.62 | 0.215 |
| Citrulline | 443.75±37.78 | 364.21±46.36 | 441.71±41.18 | 0.244 |
| Valine | 308.15±40.70 | 366.75±8.70 | 332.44±30.93 | 0.289 |
| Creatinine | 50.46±4.11 | 55.17±0.47 | 50.98±2.92 | 0.344 |
| Serine | 338.13±4.54 | 317.61±20.96 | 335.10±9.40 | 0.383 |
| Arginine | 831.16±37.19 | 815.75±29.43 | 854.60±12.62 | 0.478 |
| Tryptophan | 129.71±1.62 | 143.81±7.69 | 131.80±33.15 | 0.761 |
| TAAs | 10229.14±250.86 | 10620.22±375.75 | 10270.38±30.74 | 0.382 |
| EAAs | 2251.22±259.76^b^ | 2759.93±40.03^a^ | 2811.41±26.36^a^ | 0.060 |
| NEAAs | 6136.41±69.25^c^ | 7480.34±229.97^a^ | 7002.33±87.51^b^ | 0.006 |

Note: The same lowercase letters or no letters indicate no significant difference (*P>0.05*), and different lowercase letters indicate significant difference (*P<0.05*). TAAs, total amino acids; EAAs, essential amino acids; NEAAs: non- essential amino acids.

Supplementary Table 3 Differential metabolites in the major metabolic pathways of the three sample groups (absolute difference abundance score of all metabolic pathways ≥0.5).

| Metabolic pathways | Metabolites |
| --- | --- |
| Upregulation in the V1 group | V1vsK |
| HIF-1 signaling pathway | L-Ascorbic acid  Pyruvate |
| Carbon fixation in photosynthetic organisms | L-aspartic acid  Pyruvate |
| Alanine, aspartate and glutamate metabolism | L-aspartic acid  Pyruvate |
| Thiamine metabolism | Pyruvate  Thiamine monophosphate |
| Monobactam biosynthesis | L-aspartic acid  Pyruvate |
| Glycine, serine and threonine metabolism | L-aspartic acid  Pyruvate |
| Nicotinate and nicotinamide metabolism | L-aspartic acid  Pyruvate |
| Ascorbate and aldarate metabolism | L-Ascorbic acid  Pyruvate |
| Phosphotransferase system (PTS) | L-Ascorbic acid  Pyruvate |
| Upregulation in the V2 group | V2vsK |
| Nucleotide metabolism | His-ser  Inosine  Inosine 5'-monophosphate  Trp-Cys  Xanthosine 5'-monophosphate  Hypoxanthine  Xanthosine |
| Protein digestion and absorption | Indole  Isoleucine  L-aspartic acid  DL-tryptophan  L-methionine |
| Purine metabolism | Inosine  Inosine 5'-monophosphate  Xanthosine 5'-monophosphate  5'-phosphoribosyl-5-amino-4-imidazolecarboxamide (aicar)  Guanosine 3',5'-cyclic monophosphate  Hypoxanthine  Xanthosine |
| Metabolic pathways | Capric acid  D-proline  Glutathione, oxidized  His-ser  Indole  Inosine  Inosine 5'-monophosphate  Isoleucine  L-Ascorbic acid  L-aspartic acid |
| ABC transporters | Inosine  Isoleucine  L-aspartic acid  5-aminolevulinic acid  D-fructose  Xanthosine |
| Aminoacyl-tRNA biosynthesis | Isoleucine  L-aspartic acid  DL-tryptophan  L-methionine |
| Vitamin digestion and absorption | L-Ascorbic acid  Pyridoxal  Niacinamide |
| Cysteine and methionine metabolism | L-aspartic acid  Cysteic acid  L-methionine  S-methyl-5'-thioadenosine |
| Downregulation in the V2 group | V2vsK |
| Necroptosis  Sphingolipid signaling pathway | Cer(d18:1/18:1(9Z))  N-(octadecanoyl)sphing-4-enine-1-phosphocholine |
| Downregulation in the V1 group | V1vsV2 |
| Protein digestion and absorption | Indole  Isoleucine  L-Valine  DL-tryptophan  L-methionine |
| Mineral absorption | Isoleucine  L-Valine  DL-tryptophan  L-methionine |
| Nucleotide metabolism | Deoxyadenosine  Inosine  Trp-Cys  2'-deoxyinosine  Hypoxanthine |
| ABC transporters | Deoxyadenosine  Inosine  Isoleucine  L-Valine  2'-deoxyinosine  5-aminolevulinic acid  Choline |
| Aminoacyl-tRNA biosynthesis | Isoleucine  L-Valine  DL-tryptophan  L-methionine |
| Metabolic pathways | 2-aminoadipic acid  D-myo-inositol-1,4,5-triphosphate  Deoxyadenosine  Glutathione, oxidized  Indole  Inosine  Isoleucine  L-Anserine  L-Valine  Methionine sulfoxide  N-acetylneuraminate  Trp-Cys  Udp-n-acetylglucosamine  5-aminolevulinic acid  Choline  Cysteic acid  DL-tryptophan  Hypoxanthine  L-methionine  S-methyl-5'-thioadenosine |
| Purine metabolism | Deoxyadenosine  Inosine  2'-deoxyinosine  5'-phosphoribosyl-5-amino-4-imidazolecarboxamide (aicar)  Hypoxanthine |
| Cysteine and methionine metabolism | Methionine sulfoxide  Cysteic acid  L-methionine  S-methyl-5'-thioadenosine |
| Glucosinolate biosynthesis | Isoleucine  L-Valine  DL-tryptophan  L-methionine |
| Glycine, serine and threonine metabolism | 5-aminolevulinic acid  Choline  DL-tryptophan |
| Biosynthesis of amino acids | 2-aminoadipic acid  Isoleucine  L-Valine  DL-tryptophan  L-methionine |
| Glycerophospholipid metabolism | 1-stearoyl-2-hydroxy-sn-glycero-3-phosphocholine  Acetylcholine  Choline |
| 2-Oxocarboxylic acid metabolism | 2-aminoadipic acid  Isoleucine  L-Valine  DL-tryptophan  L-methionine |

Supplementary Table 4 Effects of valine supplementation on the AA composition and concentration of Tibetan sheep serum.

| Amino acid composition | AA content (ng/mL) | | | *p-value* |
| --- | --- | --- | --- | --- |
|  | K | V1 | V2 |  |
| Choline | 55174.26±285.52^b^ | 60567.71±285.64^a^ | 49878.52±660.28^c^ | <0.001 |
| 2-Phenylglycine | 80.07±1.77^a^ | 54.91±0.92^b^ | 51.71±0.54^b^ | <0.001 |
| Isoleucine | 5297.02±21.89^a^ | 5069.66±35.59^b^ | 4744.20±32.00^c^ | <0.001 |
| N-Methyl-aspartic acid | 61.00±0.70^a^ | 52.47±0.40^b^ | 48.64±0.10^c^ | <0.001 |
| Methionine | 4460.69±179.79^a^ | 3002.57±63.47^b^ | 2904.76±76.01^b^ | 0.002 |
| N-Acetyltyrosine | 168.27±0.10^a^ | 161.51±4.15^a^ | 131.89±2.49^b^ | 0.002 |
| Taurine | 269.67±12.26^c^ | 562.33±42.10^a^ | 454.34±1.53^b^ | 0.003 |
| Valylalanine | 2.19±0.04^a^ | 2.11±0.06^a^ | 1.79±0.03^b^ | 0.006 |
| 2-Aminoisobutyric acid | 7.77±0.46^b^ | 7.95±0.06^b^ | 10.25±0.19^a^ | 0.006 |
| α-aminobutyric acid | 5.30±0.44^b^ | 5.94±0.08^b^ | 8.99±0.66^a^ | 0.008 |
| Pipecolic acid | 51.80±1.66^a^ | 39.77±2.50^b^ | 38.07±0.02^b^ | 0.008 |
| N-Acetylneuraminic acid | 52.64±2.19^a^ | 37.69±3.67^b^ | 33.38±1.36^b^ | 0.010 |
| Glycyl-glycine | 5.43±1.05^c^ | 10.68±0.13^a^ | 8.31±0.59^b^ | 0.011 |
| N-Methylalanine | 32.16±0.39^a^ | 27.70±3.08^a^ | 18.71±0.63^b^ | 0.011 |
| Glutamic acid | 11357.51±157.10^a^ | 10420.17±284.39^b^ | 9414.94±298.45^c^ | 0.011 |
| Ornithine | 2798.54±509.60^a^ | 607.44±31.30^b^ | 1044.97±125.18^b^ | 0.011 |
| Creatinine | 4889.03±495.23^a^ | 3130.92±18.03^b^ | 2975.48±7.30^b^ | 0.012 |
| Aspartic acid | 417.09±57.43^c^ | 720.40±144.89^b^ | 1074.79±13.87^a^ | 0.012 |
| Aminoadipic acid | 193.38±31.37^b^ | 296.53±4.00^a^ | 322.92±7.74^a^ | 0.012 |
| Pyroglutamic acid | 52.45±0.04^a^ | 40.00±3.69^b^ | 38.32±0.78^b^ | 0.013 |
| Betaine | 400.93±2.99^b^ | 432.29±1.66^a^ | 418.99±7.67^a^ | 0.017 |
| Cystine | 538.38±65.09^a^ | 252.59±17.60^b^ | 481.55±42.40^a^ | 0.017 |
| Serine | 3874.10±56.44^b^ | 5517.76±367.22^a^ | 4834.73±272.24^a^ | 0.019 |
| Leucyl-glycine | 8.25±0.22^a^ | 8.61±0.43^a^ | 5.64±0.78^b^ | 0.020 |
| 2,3-Diaminopropionic acid | 30.71±1.33^b^ | 56.46±6.96^a^ | 43.97±1.93^ab^ | 0.021 |
| S-Adenosylhomocysteine | 72.45±0.99^b^ | 76.20±0.52^a^ | 75.20±0.22^a^ | 0.022 |
| 5-Aminolevulinic acid | 20.02±3.23^a^ | 15.07±0.33^ab^ | 9.69±0.16^b^ | 0.027 |
| Propionylglycine | 62.71±2.80^b^ | 99.63±10.01^ab^ | 146.36±25.02^a^ | 0.029 |
| Carnosine | 1763.41±124.40^ab^ | 2049.26±86.48^a^ | 1486.10±100.51^b^ | 0.029 |
| N-Acetylglutamic acid | 30.63±1.84^b^ | 36.42±1.20^a^ | 30.64±0.38^b^ | 0.032 |
| Anserine | 208.70±16.00^ab^ | 228.44±0.01^a^ | 182.18±1.57^b^ | 0.035 |
| Homoserine | 3029.44±266.42^ab^ | 2487.84±272.50^b^ | 3757.29±227.17^a^ | 0.036 |
| N-Acetylaspartic acid | 16.17±1.04^b^ | 17.37±2.81^b^ | 24.31±0.96^a^ | 0.038 |
| N6-Acetyllysine | 33.94±4.75^b^ | 41.91±0.29^ab^ | 46.63±0.25^a^ | 0.042 |
| Threonine | 4022.17±391.83^ab^ | 3271.22±394.24^b^ | 4884.41±308.88^a^ | 0.049 |
| γ-Glutamyl-phenylalanine | 8.54±1.11^b^ | 13.42±1.15^a^ | 10.16±1.33^ab^ | 0.057 |
| 4-Acetamidobutanoic acid | 16.81±3.87^a^ | 7.27±2.07^b^ | 8.04±1.06^b^ | 0.060 |
| N-Isovaleroylglycine | 29.34±0.39^b^ | 45.06±3.48^a^ | 34.00±6.05^ab^ | 0.063 |
| Proline | 460.99±37.64^b^ | 492.56±45.46^ab^ | 592.60±23.11^a^ | 0.073 |
| Phenylacetylglutamine | 58.55±8.32^ab^ | 49.89±1.10^b^ | 69.26±3.24^a^ | 0.074 |
| Hydroxyproline | 2427.48±328.67^ab^ | 2201.09±93.36^b^ | 2953.48±104.37^a^ | 0.074 |
| Sarcosine | 5106.49±81.52^ab^ | 4557.41±353.85^b^ | 5330.81±69.15^a^ | 0.075 |
| β-Alanine | 6925.07±46.09^ab^ | 6134.00±476.49^b^ | 7109.74±74.49^a^ | 0.076 |
| 3-Hydroxyhippuric acid | 39.41±3.74^a^ | 38.72±3.49^ab^ | 27.39±3.85^b^ | 0.079 |
| 2,6-Diaminopimelic acid | 34.73±0.13^b^ | 35.06±0.04^ab^ | 40.50±3.10^a^ | 0.080 |
| Creatine | 1612.17±131.99^a^ | 1412.10±11.36^ab^ | 1354.76±21.14^b^ | 0.088 |
| Cystathionine | 977.79±114.04^b^ | 1102.16±64.71^ab^ | 1242.28±16.54^a^ | 0.089 |
| Cis-4-Hydroxy-D-proline | 2407.07±291.78^ab^ | 2221.04±96.59^b^ | 2861.01±137.77^a^ | 0.094 |
| γ-Glutamylalanine | 250.78±61.99^b^ | 304.08±28.23^ab^ | 455.00±86.27^a^ | 0.098 |
| Glycyl-phenylalanine | 2.15±0.58 | 1.14±0.21 | 1.15±0.10 | 0.105 |
| Tyrosine | 7392.12±564.19^a^ | 6664.54±693.10^ab^ | 5696.27±211.43^b^ | 0.107 |
| Argininosuccinic acid | 147.48±5.82 | 136.49±1.99 | 142.06±0.29 | 0.117 |
| γ-Glutamyl-valine | 66.61±7.42 | 53.80±8.49 | 72.11±4.97 | 0.165 |
| Phosphoserine | 138.78±0.34 | 138.71±0.19 | 139.23±0.09 | 0.185 |
| Dimethylglycine | 15.04±0.23 | 14.57±3.50 | 10.61±0.89 | 0.213 |
| Histidine | 2315.09±113.64 | 2507.65±103.91 | 2428.98±10.68 | 0.242 |
| Methionine sulfoxide | 634.96±140.87 | 474.26±36.07 | 475.99±21.35 | 0.242 |
| Asparagine | 2843.37±50.86 | 2824.02±116.53 | 2968.14±10.60 | 0.252 |
| Trimethylamine-N-oxide | 4.54±1.32 | 5.16±1.35 | 6.73±0.32 | 0.269 |
| 4-Guanidinobutanoic acid | 286.27±13.91 | 279.48±20.69 | 309.18±10.05 | 0.279 |
| 3-Methoxytyrosine | 135.84±0.88 | 131.20±1.65 | 131.14±4.58 | 0.308 |
| Citrulline | 7747.13±249.04 | 8038.00±407.69 | 7339.44±474.93 | 0.332 |
| Cysteine | 3383.68±655.95 | 2524.55±518.06 | 2837.77±61.26 | 0.334 |
| Glycylleucine | 21.86±1.74 | 20.57±0.05 | 20.36±0.01 | 0.392 |
| Glycine | 23225.73±1971.43 | 23499.36±90.82 | 21907.48±123.31 | 0.435 |
| 5-Aminopentanoic acid | 21.06±7.19 | 21.94±2.36 | 28.51±6.72 | 0.473 |
| Aminocaproic acid | 1606.13±232.73 | 1403.64±27.44 | 1493.60±127.79 | 0.505 |
| Norleucine | 9294.85±446.17 | 9295.19±795.01 | 9876.83±19.14 | 0.522 |
| 1-Methylhistidine | 1169.66±409.95 | 1245.47±165.65 | 1479.66±91.85 | 0.538 |
| Leucine | 6199.25±10.88 | 6081.64±241.14 | 6019.16±177.80 | 0.622 |
| Valine | 12367.50±2118.45 | 8264.08±9972.47 | 13920.59±34.28 | 0.653 |
| Alanine | 9056.77±40.61 | 9292.06±770.43 | 9450.67±214.85 | 0.720 |
| Glutamine | 9216.79±2346.54 | 8522.78±567.53 | 8194.15±197.18 | 0.774 |

Note: The same lowercase letters or no letters indicate no significant difference (*P>0.05*), and different lowercase letters indicate significant difference (*P<0.05*).

Supplementary Table 5 Effects of valine supplementation on the AA composition and concentration of Tibetan sheep rumen fluid.

| Amino acid composition | Amino acid content (ng/mL) | | | *p-value* |
| --- | --- | --- | --- | --- |
|  | K | V1 | V2 |  |
| Homocitrulline | 913.78±8.55^b^ | 649.40±51.29^c^ | 1402.35±39.20^a^ | <0.001 |
| Aspartic acid | 8935.61±306.50^c^ | 10859.70±284.74^b^ | 12339.77±141.86^a^ | 0.002 |
| Propionylglycine | 108.55±4.15^b^ | 74.68±5.95^c^ | 150.76±9.61^a^ | 0.004 |
| 2,6-Diaminopimelic acid | 258.10±9.25^a^ | 197.38±9.51^b^ | 272.36±1.69^a^ | 0.005 |
| Aspartylphenylalanine | 32.88±1.13^b^ | 24.49±1.70^c^ | 46.12±3.76^a^ | 0.007 |
| Hydroxyproline | 270.48±14.87^a^ | 97.23±19.89^b^ | 120.18±27.67^b^ | 0.007 |
| N6-Acetyllysine | 265.76±105.08^b^ | 342.09±3.29^b^ | 773.56±21.86^a^ | 0.007 |
| N-Acetylglutamic acid | 1882.43±16.68^a^ | 1149.62±45.48^b^ | 1965.00±172.30^a^ | 0.007 |
| Creatinine | 95.18±15.79^a^ | 23.55±0.34^b^ | 18.54±10.55^b^ | 0.010 |
| N-Acetylneuraminic acid | 11.79±3.71^b^ | 13.69±0.22^b^ | 26.39±0.41^a^ | 0.012 |
| Homoserine | 7183.18±954.85^b^ | 11981.23±289.63^a^ | 15206.06±1716.16^a^ | 0.014 |
| L-Theanine | 3.53±0.01^b^ | 4.60±0.38^a^ | 5.05±0.09^a^ | 0.015 |
| 1-Methylhistidine | 20.99±2.64^a^ | 9.87±0.96^b^ | 9.74±1.66^b^ | 0.015 |
| Glycine | 29659.77±2910.22^a^ | 20558.41±1174.97^b^ | 31620.38±356.77^a^ | 0.017 |
| Cis-4-Hydroxy-D-proline | 406.38±35.75^a^ | 227.46±30.37^b^ | 326.26±19.80^a^ | 0.020 |
| N-α-acetyllysine | 387.10±21.00^b^ | 301.26±37.60^b^ | 654.50±93.83^a^ | 0.020 |
| Isoleucine | 2498.55±127.66^b^ | 2526.40±168.80^b^ | 3131.11±11.96^a^ | 0.023 |
| γ-Aminobutyric acid | 292.36±50.43^a^ | 137.05±4.92^b^ | 145.81±18.47^b^ | 0.026 |
| β-Alanine | 19275.02±47.09^a^ | 14648.34±231.14^b^ | 18975.55±1625.46^a^ | 0.028 |
| Asparagine | 176.92±10.86^a^ | 74.95±19.70^b^ | 101.69±25.17^b^ | 0.028 |
| Leucine | 2917.92±175.78^b^ | 2802.77±39.28^b^ | 3542.71±185.92^a^ | 0.030 |
| Choline | 60919.55±5390.47^b^ | 83282.42±41.06^a^ | 84996.70±7594.52^a^ | 0.035 |
| Histidine | 171.96±12.62^b^ | 198.84±15.12^b^ | 418.60±92.14^a^ | 0.036 |
| Valine | 26261.21±431.03^a^ | 2735.43±708.44^b^ | 36031.68±12410.98^a^ | 0.040 |
| γ-Glutamyl-phenylalanine | 418.64±94.77^a^ | 187.98±6.27^b^ | 226.14±3.96^b^ | 0.046 |
| 5-Aminolevulinic acid | 77.00±22.80^b^ | 85.32±14.57^b^ | 281.53±84.40^a^ | 0.046 |
| Leucyl-glycine | 54.20±2.33^ab^ | 35.83±18.59^b^ | 83.82±0.57^a^ | 0.047 |
| 2,3-Diaminopropionic acid | 124.86±26.20^a^ | 51.26±1.50^b^ | 128.77±23.04^a^ | 0.051 |
| Argininosuccinic acid | 167.29±46.08^b^ | 277.29±0.40^a^ | 275.19±21.55^a^ | 0.053 |
| Methionine sulfoxide | 12289.95±355.91^a^ | 8534.89±1220.71^b^ | 12568.12±1500.10^a^ | 0.064 |
| Proline | 3948.02±364.23^ab^ | 3493.75±137.98^b^ | 4528.57±235.68^a^ | 0.065 |
| Sarcosine | 12258.94±100.05^a^ | 9844.14±447.04^b^ | 12080.48±1121.66^a^ | 0.069 |
| γ-Glutamyl-methionine | 135.23±7.50^ab^ | 108.95±7.07^b^ | 143.87±13.31^a^ | 0.074 |
| Alanine | 22396.63±319.01^a^ | 18043.00±851.04^b^ | 22013.75±2048.13^ab^ | 0.075 |
| Taurine | 50.79±4.44^a^ | 43.16±0.37^ab^ | 41.14±1.83^b^ | 0.078 |
| α-aminobutyric acid | 237.11±69.92^a^ | 106.66±0.37^b^ | 121.08±7.12^ab^ | 0.086 |
| Valylalanine | 9.62±0.54 | 8.51±2.81 | 15.16±2.45 | 0.102 |
| Tryptophan | 6010.85±646.88^ab^ | 5043.75±320.04^b^ | 6705.85±532.60^a^ | 0.106 |
| 4-Guanidinobutanoic acid | 576.94±40.02 | 498.41±28.46 | 614.86±40.62 | 0.106 |
| Kynurenate | 92.13±15.98 | 60.01±8.09 | 79.80±0.08 | 0.113 |
| N-Acetylaspartic acid | 76.34±15.75 | 83.18±18.22 | 119.49±16.06 | 0.148 |
| N-Acetyltyrosine | 469.79±51.82 | 517.84±76.95 | 679.48±110.71 | 0.166 |
| Glycyl-phenylalanine | 27.56±4.79 | 27.35±7.75 | 39.40±0.78 | 0.169 |
| Threonine | 15183.00±1862.94 | 14638.02±705.51 | 17987.46±1339.02 | 0.171 |
| Cysteine | 3463.11±232.98 | 2593.66±9.79 | 3494.34±650.53 | 0.176 |
| 5-Hydroxy-tryptophan | 35.05±1.47 | 31.55±0.72 | 35.21±2.37 | 0.186 |
| Creatine | 95.75±59.82 | 10.31±8.07 | 21.78±25.20 | 0.191 |
| Serine | 17404.53±591.05 | 14421.42±569.08 | 16949.64±2110.95 | 0.191 |
| γ-Glutamyl-valine | 1846.24±34.50 | 1374.52±249.40 | 1982.42±389.45 | 0.203 |
| Glycylvaline | 67.21±4.15 | 74.24±1.97 | 194.81±107.68 | 0.217 |
| Citrulline | 5601.95±660.94 | 4748.03±480.95 | 5744.37±77.77 | 0.223 |
| Anserine | 64.78±2.45 | 86.51±14.03 | 65.22±14.71 | 0.257 |
| Pyroglutamic acid | 1252.77±345.98 | 835.48±24.26 | 1269.43±237.89 | 0.275 |
| 5-Aminopentanoic acid | 1961.78±139.41 | 1974.16±404.17 | 2396.74±32.32 | 0.280 |
| Kyotorphin | 3.68±0.21 | 3.47±0.12 | 3.84±0.22 | 0.287 |
| Pipecolic acid | 1256.39±359.54 | 835.34±30.21 | 1265.12±242.49 | 0.291 |
| (S)-β-Aminoisobutyric acid | 18.71±0.51 | 29.49±15.15 | 35.41±0.40 | 0.297 |
| O-Succinyhomoserine | 64.13±1.74 | 55.75±2.76 | 70.25±13.28 | 0.321 |
| Nα-Acetyl-L-arginine | 1.32±1.82 | 4.80±0.43 | 2.72±2.93 | 0.349 |
| Tyrosine | 11605.98±1389.03 | 9536.27±959.87 | 11786.72±2099.24 | 0.394 |
| Glutamine | 1746.49±92.74 | 2202.86±418.50 | 1768.35±436.80 | 0.448 |
| Glutamic acid | 17296.24±472.91 | 15654.42±1073.28 | 17125.10±1834.08 | 0.456 |
| Phosphoserine | 207.09±39.69 | 224.36±9.43 | 200.02±5.46 | 0.623 |
| Ornithine | 159.20±125.98 | 134.37±5.76 | 278.99±223.25 | 0.628 |
| Glycylleucine | 48.95±3.97 | 48.12±13.05 | 56.47±8.88 | 0.660 |
| Glycyl-glycine | 62.31±2.42 | 71.53±25.77 | 80.31±33.69 | 0.781 |
| L-Tyrosine methyl ester | 15.84±1.13 | 14.98±0.61 | 15.67±2.05 | 0.818 |
| N-Methylalanine | 249.40±136.21 | 201.11±4.26 | 225.82±14.27 | 0.839 |
| 2-Aminoisobutyric acid | 137.03±76.68 | 110.00±2.41 | 124.10±9.69 | 0.841 |

Note: The same lowercase letters or no letters indicate no significant difference (*P>0.05*), and different lowercase letters indicate significant difference (*P<0.05*).
